# Fecal identification markers impact the feline fecal microbiota

**DOI:** 10.3389/fvets.2023.1039931

**Published:** 2023-02-08

**Authors:** Nora Jean Nealon, Alexandra Wood, Adam J. Rudinsky, Hannah Klein, Matthew Salerno, Valerie J. Parker, Jessica M. Quimby, James Howard, Jenessa A. Winston

**Affiliations:** ^1^Department of Veterinary Clinical Sciences, College of Veterinary Medicine, Comparative Hepatobiliary and Intestinal Research Program, The Ohio State University, Columbus, OH, United States; ^2^Department of Veterinary Clinical Sciences, College of Veterinary Medicine, The Ohio State University, Columbus, OH, United States

**Keywords:** 16S rRNA, fecal microbiota, feline microbiota, fecal sample, veterinary medicine, fecal marker, amplicon sequencing

## Abstract

Fecal diagnostics are a mainstay of feline medicine, and fecal identification markers help to distinguish individuals in a multi-cat environment. However, the impact of identification markers on the fecal microbiota are unknown. Given the increased interest in using microbiota endpoints to inform diagnosis and treatment, the objective of this study was to examine the effects of orally supplemented glitter and crayon shavings on the feline fecal microbiota (amplicon sequencing of 16S rRNA gene V4 region). Fecal samples were collected daily from six adult cats that were randomized to receive oral supplementation with either glitter or crayon for two weeks, with a two-week washout before receiving the second marker. No adverse effects in response to marker supplementation were seen for any cat, and both markers were readily identifiable in the feces. Microbiota analysis revealed idiosyncratic responses to fecal markers, where changes in community structure in response to glitter or crayon could not be readily discerned. Given these findings, it is not recommended to administered glitter or crayon shavings as a fecal marker when microbiome endpoints are used, however their clinical use with other diagnostics should still be considered.

## 1. Introduction

Gastrointestinal conditions are a leading cause of veterinary examinations for feline patients (1). As clinical knowledge of gastrointestinal diseases expands, the role of the gut microbiota in feline health vs. disease is becoming increasingly investigated (2–4). The feline gut microbiota, which consists of prokaryotic, eukaryotic, and viral communities, has been shown to modulate host metabolism, nutrient digestibility, immune function, and susceptibility to chronic and infectious diseases (5, 6). Given the increasing recognition of the gut microbiota on feline health, ongoing efforts have harnessed the fecal microbiota to assist with the diagnosis, treatment, and medical understanding of diseases. In the feline gut, dysbiosis, a disruption in microbiota composition and function, has been linked with several feline diseases including diabetes mellitus, inflammatory bowel disease, and chronic kidney disease (7–9).

Sample collection is a key consideration in any microbiota study. Fecal samples are commonly used to evaluate gut microbial communities because of their ease of acquisition. However, unlike other species, fecal collection in cats is limited by husbandry needs (e.g., group housing) and feline elimination behaviors (e.g., preference for privacy, fastidious environments, ability to hide their feces, variable day-to-day timing of defecation, and lower defecation frequency) (10, 11). These differences create a challenge in identification of an individual cat's feces in multi-cat households and group housing research facilities. Oral administration of fecal identification markers, such as crayon shavings and glitter, have been routinely used to aid in accurate fecal collection, where previous studies support their ease of administration, identification, and discrimination between individual cat fecal samples (11–13). However, it is unknown how these exogenous fecal markers impact the gut microbial community structure.

The objective of this study was to determine whether orally administered fecal identification markers (glitter and crayon shavings) alter the fecal microbial community structure in healthy cats. We hypothesized that oral administration of crayon shavings and/or glitter would not alter the fecal microbiota community in healthy, adult purpose breed research cats.

## 2. Materials and methods

### 2.1. Animals and housing

**Figure 1:**
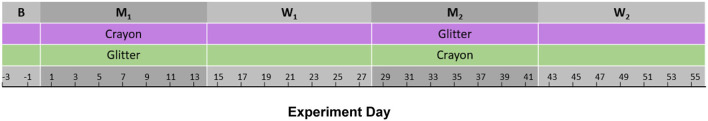
Experimental Timeline. Cats were randomly assigned to one of two groups, who would receive oral administration of crayon shavings during M_1_ followed by glitter during M_2_ (group CG, cats 1–3; *denoted in purple*) or glitter followed by crayon shavings administration (group GC, cats 4–6, *denoted in green*), with three cats in each group. B, Baseline; M_1_, Marker 1; W_1_, Washout 1; M_2_, Marker 2; W_2_, Washout 2.

Six healthy, adult, purpose-bred, research cats (*n* = 3 castrated males, *n* = 3 ovariohysterectomized females) were included in a randomized cross-over study (Figure 1). All cats were group housed in a research facility for several months prior to study initiation. Cats were deemed healthy by physical examination performed by two board-certified veterinary internal medicine specialists and comprehensive bloodwork (complete blood count, serum biochemistry panel, urinalysis, and fecal flotation). Access to food and clean drinking water were provided *ad libitum*.

During a three-week acclimatization period prior to the beginning of this study, all cats were transitioned from their previous commercial laboratory feline dry diet (Lab Feline Diet #5003 dry formulation, Cincinnati Lab Supply Inc.) to Nestle Purina Pro Plan Complete Essentials Chicken and Rice canned diet. This transition occurred so that each fecal marker could be blended into the diet for daily oral administration. Each cat was fully eating this canned diet for 36 days prior to the start of this current study. Cats were fed twice a day based on their resting energy requirement, where weekly body weights were used to adjust daily caloric intake to maintain their weight. All cats were maintained on the same canned diet for the remainder of the study. The nutrient profile of both diets is provided in Supplementary Table 1. Over the course of the study, Nestle Purina fecal score, calorie intake, daily observations, and presence of the fecal identification marker were recorded daily. An ideal fecal score was defined as two out of seven. The Nestle Purina fecal scoring system used in this study is summarized in Supplementary Table 2 (14). Weight was monitored through weekly assessments and recorded in each animal's medical record. All protocols were approved by The Ohio State University Institutional Animal Care and Use Committee (2017A00000093-R1). Following this study, all cats remained under the care of University Laboratory Animal Services and were ultimately adopted into client-owned households.

### 2.2. Fecal identification marker administration and fecal collection

Figure 1 shows the experimental timeline over which cats received each of the two fecal markers. Cats were randomized into two groups (*n* = 3 per group) that received oral administration of crayon shavings followed by glitter (denoted as CG group) or glitter followed by crayon shavings administration (denoted as GC group). Fecal sample collection was performed overnight when cats were individually housed in kennels according to the standard management protocol. Fecal samples were collected daily from each cat over a 3-day baseline period. Groups were then orally administered either glitter or crayon shavings mixed into their canned food for 14 days (M_1_ = Marker 1 phase). Crayon shavings and glitter administered orally were non-toxic and included Crayola paraffin wax-based crayons and Bakell edible non-toxic decorative glitter. Marker 1 phase was followed by a 14-day washout period (W_1_ = Washout 1) where no cat received oral fecal identification markers. Group cross-over was performed for glitter or crayon shaving administration for 14 days (M_2_ = Marker 2 phase), followed by another 14-day washout (W_2_ = Washout 2). Each cat was initially administered a 2.5 mL portion (1/2 teaspoon) of glitter or crayon shavings per day mixed into canned food. The dose was increased to 5 mL (1 teaspoon) on day 4 (M_1_ phase) to enhance visualization in feces and this higher dose was maintained for the remainder of the study's marker phases. Naturally voided fecal samples from individual cats were collected from litterboxes daily during the study period. The litter substrate was fine wood shavings. Based on previously published protocols, feces was collected each morning from overnight defecation and were <12–14 h old at the time of collection (15). Feces was aliquoted (500–1,000-gram aliquots) into cryovials within 1 h of collection and frozen at −80°C for future analysis.

### 2.3. DNA extraction and amplicon sequencing

Fecal samples underwent DNA extraction and quantification at University of Michigan Microbiome Core. A MagAttract PowerMicrobiome kit (Qiagen, Germantown, MD, USA) extracted DNA from each fecal sample following manufacturer instructions. An Eppendorf EpMotion liquid handling system (Eppendorf, Enfield, CT, USA) aliquoted each sample during DNA extraction. Following extraction, a Quant-iT PicoGr4een dsDNA Assay fluorometric kit (ThermoFisher Scientific, Waltham, MA, USA) quantified 1 μL aliquots of each sample for use in 16s rRNA gene PCR amplification.

The V4 region of the 16s rRNA gene was amplified from each sample using a dual indexing approach (16). Genes were amplified using primers 515f and 806r (17). PCR master mix contained 2 μL 10x AccuPrime PCR Buffer II (ThermoFisher Scientific), 11.85 μL double-distilled water, 0.15 μL AccuPrime High Fidelity Taq Polymerase (ThermoFisher Scientific), 1 μL of each DNA sample, and 5 μL of a 4 μM solution of each primer. Following an initial 120 s at 95°C, 30 PCR amplification cycles occurred under the following parameters: denaturation at 95°C for 20 s, annealing at 55°C for 15 s, then 72°C for 900 s. PCR products were incubated at 4°C until further analysis. An E-Gel 96 with 2% SYBR Safe DNA Gel Stain (ThermoFisher Scientific) was used to visualize PCR products. Sterile extraction reagents and master mix served as negative quality controls throughout DNA processing and amplification.

Amplicon libraries normalization was performed using a SequalPrep Normalization Plate Kit (Life technologies, Carlsbad, CA, USA) following manufacturer protocols for sequential elution. A Kapa Biosystems Library Quantification kit for Illumina platforms (Kapa Biosystems, Wilmington, MA, USA) determined the concentration of the pooled samples so that the final library size consisted of equimolar amounts of each sample normalized to the lowest sample concentration. An Agilent Bioanalyzer High Sensitivity DNA analysis kit (Santa Clara, CA, USA) determined the amplicon sizes in the pooled library.

Paired-end, *de-novo* amplicon sequencing was performed on an Illumina MiSeq platform with a MiSeq reagent kit (Illumina, San Diego, CA, USA) using V2 chemistry with 500 cycles for 2 nM or 4 nM libraries according to manufacturer protocols with slight modifications as previously described (16). The final library load concentration was 5.5 nM with a 15% PhiX spike (Illumina) to create diversity. All Illumina sequencing reagents were prepared according to Illumina use protocols for the MiSeq personal sequencer system (16). Custom read 1, custom read 2, and index primers were added to the Illumina reagent cartridge prior to sequencing. FASTQ files were generated for each paired-end read. Sterile extraction reagents and master mix served as negative quality controls throughout sample sequencing.

### 2.4. Fecal microbiota analysis

Amplicon (V4 16S rRNA gene) sequence analysis was performed using R Studio (Version 2022.07.1, Build 554) (18). Following importation into R, each pair-end reads were assembled into contigs, trimmed, filtered, and converted into amplicon sequence variants (ASV) via the DADA2 pipeline (19). Prior to taxonomic classification, ASVs <250 base pairs in length or >256 base pairs in length were removed along with chimera sequences. Taxonomy was assigned to ASVs using the Silva 16s rRNA Sequence Database (Version 138.1) (20). Raw ASV counts within taxonomy tables were normalized to percent relative abundance and ASVs contributing to < 1% of the sample percent relative abundance were removed from downstream analysis. All taxonomic classification and taxonomy table generation were performed using the Phyloseq package (Version 1.40.0) in R studio (21). Supplementary material 1 shows, for each sequence, the number of reads and mean quality scores both pre and post filtering, the number of reads during each processing step of DADA2 ASV generation, and the number of phyla and families for each sample before and after filtering those with <1% percent relative abundance.

Alpha and beta diversity analysis were performed using the Phyloseq (Version 1.40.0) and Vegan (Version 2.6-2) packages in R studio (21, 22). Alpha diversity metrics were calculated for each sample using Shannon, Inverse Simpson, and observed ASVs matrices. Alpha diversity metrics were visualized in GraphPad Prism (Version 9.3.1 for MAC OS X, GraphPad Software, LLC, La Jolla, CA, USA). Beta diversity was calculated using a Bray-Curtis dissimilarity algorithm, where samples were visualized in R studio using a non-metric multidimensional scaling (NMDS) approach. NMDS stress values <0.2 were considered acceptable (23). Cat 6 had a single outlier (day 43 during W_2_ when hairball vomiting reported) that was removed from the individual NMDS plot to allow for appropriate visualization of data.

### 2.5. Statistical analysis

Daily feces collected from each individual cat were included in the statistical analysis of clinical (i.e., weight and fecal score) and microbiome endpoints. A linear mixed effects model with a Bonferroni multiple comparisons correction was used to examine the impact of treatment group (GC vs. CG) and experimental phase (independent variables) on body weight (dependent variable). A Shapiro-Wilk test confirmed that body weights were normally distributed for the GC (*p* = 0.63) and CG (*p* = 0.45) treatment groups. A Mann-Whitney test with a Benjamini-Hochberg correction was used to assess the impact of treatment group and experimental phase (independent variables) on fecal score (dependent variable). A Shapiro-Wilk test indicated that fecal scores were normally distributed within group GC (*p* = 0.85) but not within group CG (*p* = 0.0116). For each alpha diversity matrix, a Mann-Whitney test with a Benjamini-Hochberg correction compared fecal identification marker order [i.e., glitter then crayon (GC group) vs. crayon then glitter (CG group)] at each study day. A pairwise PERMANOVA analysis (permutational multivariate analysis of variance) with a Benjamini-Hochberg *post-hoc* correction compared microbial community structure between all cats and across experimental phases for individual cats using the Phyloseq and vegan packages in R studio. Differential abundance of microbial phyla and families was performed for each individual cat using the Deseq2 package in R studio where parameters were set to the following: Test = Wald, FitType = Parametric, Cook's Cutoff = FALSE (i.e., not applied to the dataset), and Benjamini-Hochberg *post-hoc* correction (24). The following comparisons were assessed using Deseq2: baseline vs. M_1_, baseline vs. W_1_, baseline vs. M_2_, baseline vs. W_2_, M_1_ vs. W_1_, M_1_ vs. M_2_, M_1_ vs. W_2_, W_1_ vs. M_2_, W_1_ vs. W_2_, and M_2_ vs. W_2_. For all analyses, significance was defined as *p* < 0.05 following *post-hoc* correction. For Cat 6, the Day 43 fecal sample was included in all statistical analyses.

#### 2.5.1. Data availability

All sequences included in the analysis herein are publicly available via the National Center for Biotechnology Information Sequence Read Archive (NCBI SRA) via the following BioProject identification number: PRJNA862255.

## 3. Results

### 3.1. Body weight, fecal scores, and adverse event reporting

**Figure 2:**
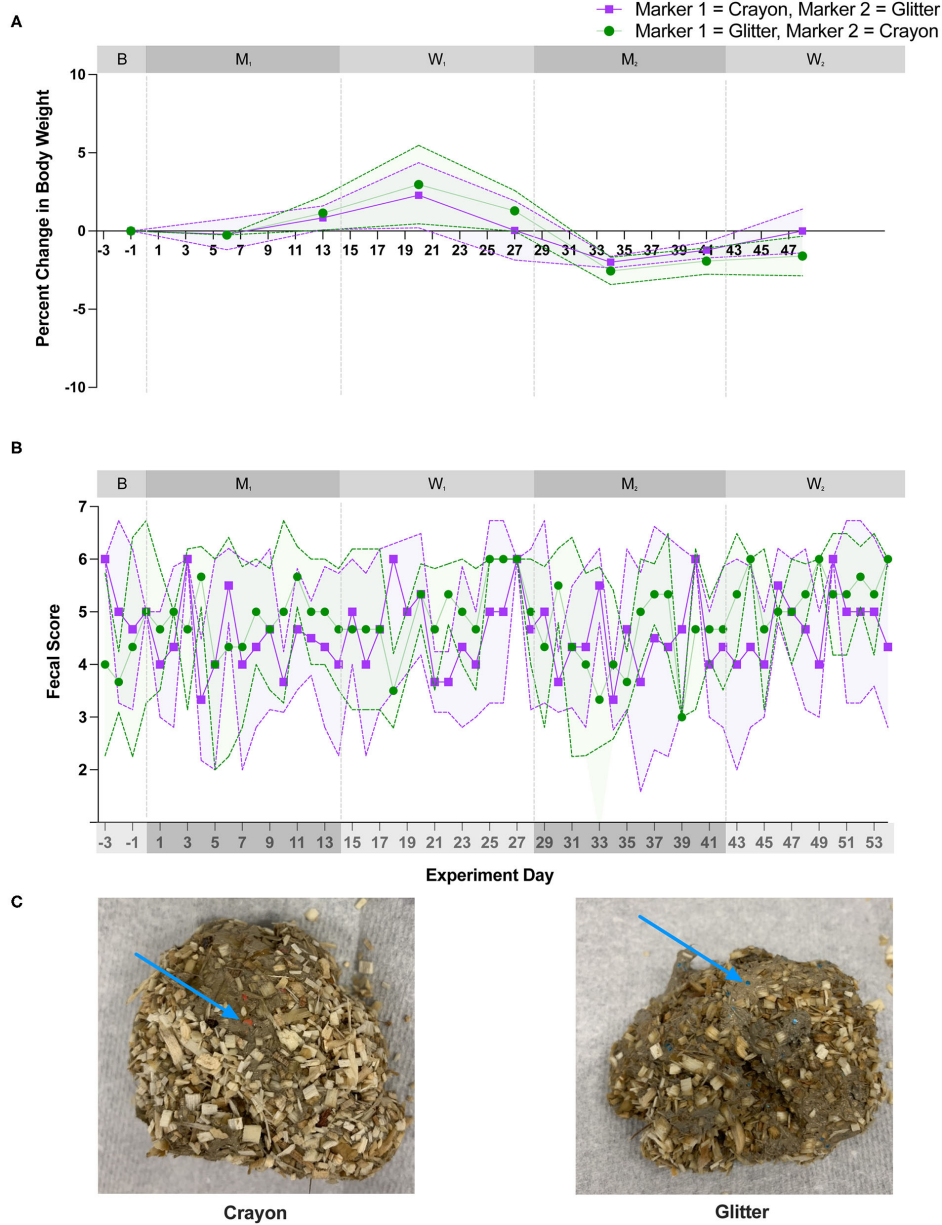
There was no significant difference in weight or fecal scores with oral administration of fecal identification markers. **(A)** Mean percent change body weight and standard deviation and **(B)** mean fecal score and standard deviation over 56-day experiment. Points represent the mean value for group GC and group CG at each day weight and/or fecal scores were measured. Dotted lines bordering each shaded region represent the standard deviation. Statistical significance was determined using a linear mixed effect model for body weight changes and a Mann-Whitney test for fecal score changes when comparing marker order treatment groups [i.e., glitter then crayon (group GC) vs. crayon then glitter (group CG) at each experimental phase]. Significance was defined as *p* < 0.05 for both analyses following Bonferroni multiple comparisons correction (body weights) and a Benjamini-Hochberg correction (fecal scores). **(C)** Crayon shavings (red) and glitter (blue) were readily visible in cat feces (denoted by blue arrow).

All cats consumed their resting energy requirement for the entire duration of the study. Figure 2 shows cat body weights and fecal scores over the study duration. For all cats, body weights remained stable across each experimental phase. The mean percent change in body weight in each group of cats was +/– 3% from baseline throughout the 56-day long study (Figure 2A). Fecal scores increased during the 3-week acclimatization period when the diet was transitioned from dry to canned food (data not shown). After this transition, fecal scores remained consistently higher than the ideal score of 2/7 during the 56-day study period (Figure 2B). There was no significant change in mean fecal score between groups during the baseline, M_1_, W_1_, and M_2_ experimental phases. However, during W_2_, group CG experienced a statistically significant increase in fecal score compared to group GC (*p* = 0.0282; Figure 2B). Glitter and crayon shavings were readily identifiable in feces from all cats (Figure 2C). During the washout phases, crayon shavings were visible in the feces of all cats for 1–2 days and glitter was visible for 1–4 days after each fecal marker was removed from the canned food.

Two cats experienced vomiting during the study. Cat 4 vomited a total of five times, once on day 37 (M_2_) and four times during W_2_ phase (day 42, 43, 45, and 46). On all occasions, the vomitus was described as a hairball. Cat 6 vomited once on day 54 (W_2_) which was also described as a hairball. No additional adverse events were observed for any of the cats during this study.

### 3.2. Alpha and beta diversity metrics

**Figure 3:**
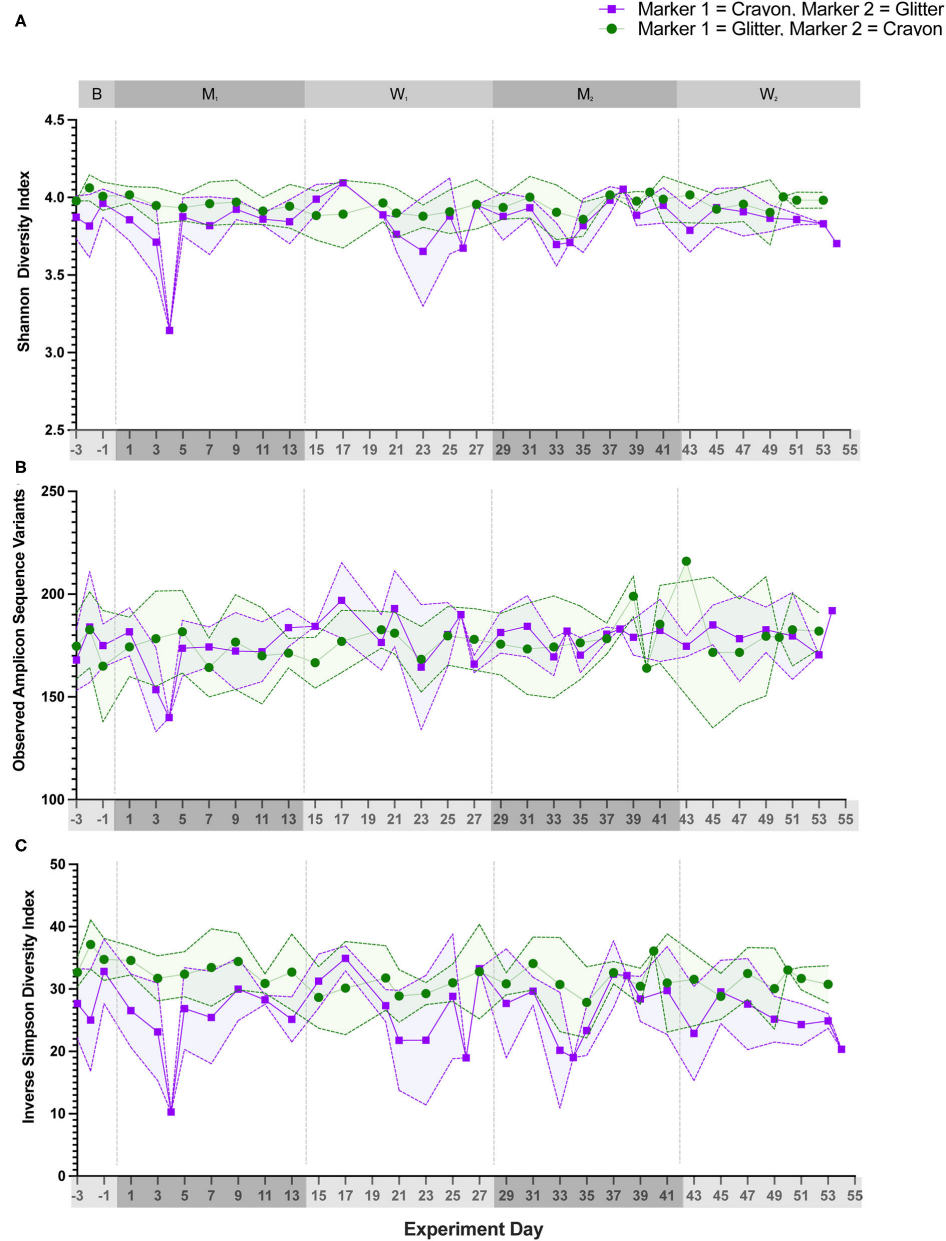
Alpha diversity metrics did not significantly differ between treatment groups during any study day. Three alpha diversity metrics were calculated for each fecal sample. **(A)** Shannon diversity index, **(B)** Observed number of Amplicon Sequencing Variants (ASVs), and **(C)** Inverse simpson diversity index. Statistical significance was determined using a Mann-Whitney test to compare treatment groups [i.e., glitter then crayon (GC group) vs. crayon then glitter (CG group)] at each study day. Significance was defined as *p* < 0.05 for both analyses following a Benjamini-Hochberg correction. Points represent the mean score for each day, and the dotted lines bordering each shaded region represent the standard deviation.

**Figure 4:**
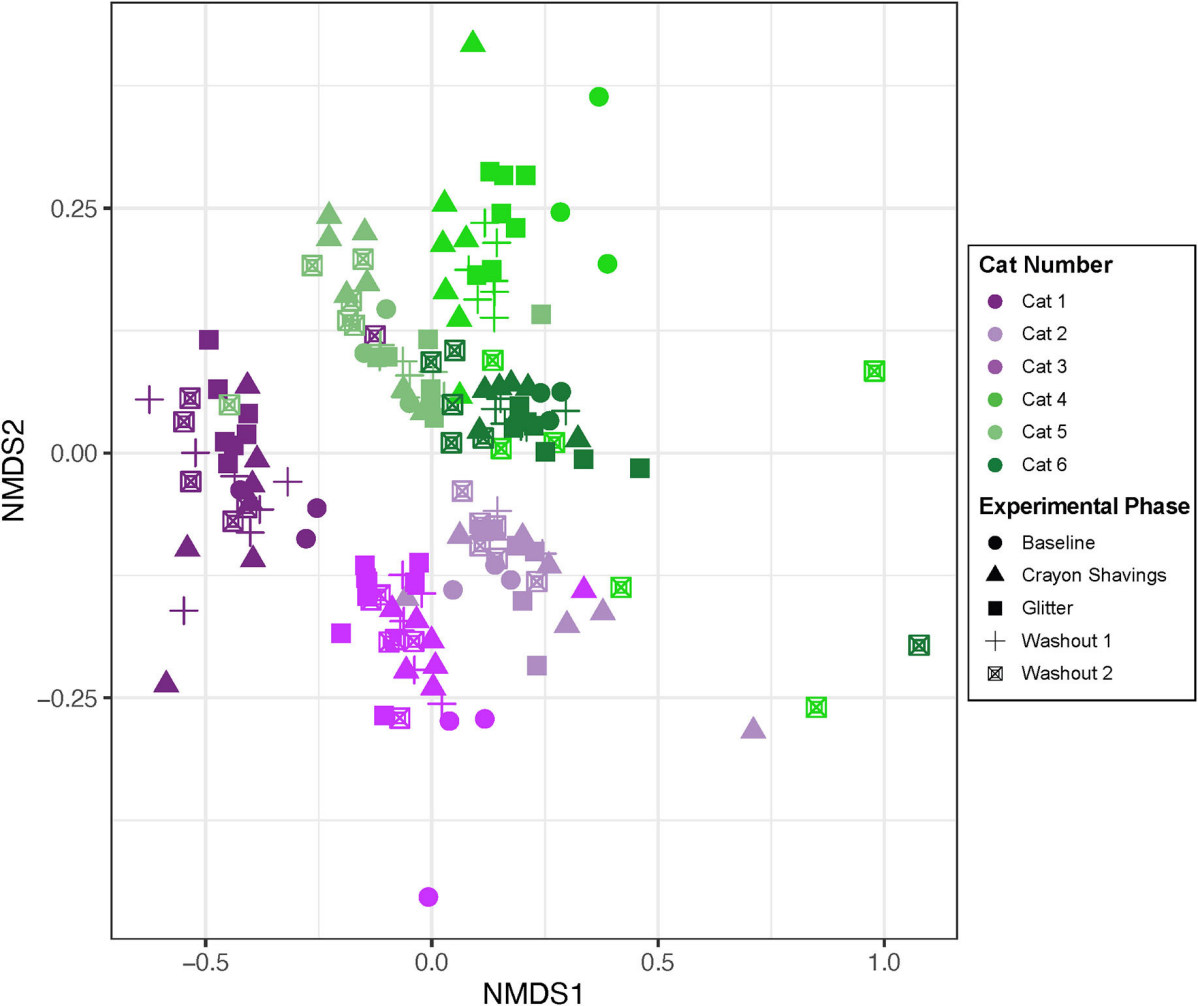
Fecal sample microbiota compositions cluster by individual cats. NMDS ordination was calculated with Bray-Curtis dissimilarity algorithm on ASVs from fecal samples. Statistical significance of microbial community structure between all cats was determined with pairwise PERMANOVA analysis with a Benjamini-Hochberg *post-hoc* correction. Each point represents an individual fecal sample from one cat. CG group is denoted in purple (Cats 1, 2, and 3). GC group is denoted in green (Cats 4, 5, and 6). Experimental phase is represented by a different symbol as denoted in the legend.

Alpha diversity (Shannon Diversity Index, observed ASVs, and Inverse Simpson Diversity Index) did not differ significantly between treatment groups during any experimental phase (Figure 3; *p* = 0.1000 to *p* < 0.9999). Beta diversity analysis for all cats revealed that fecal samples from individual cats clustered more closely to other fecal samples from the same cat independent of experimental phase (Figure 4; PERMANOVA, *p* < 0.0001 for all cat comparisons).

**Figure 5:**
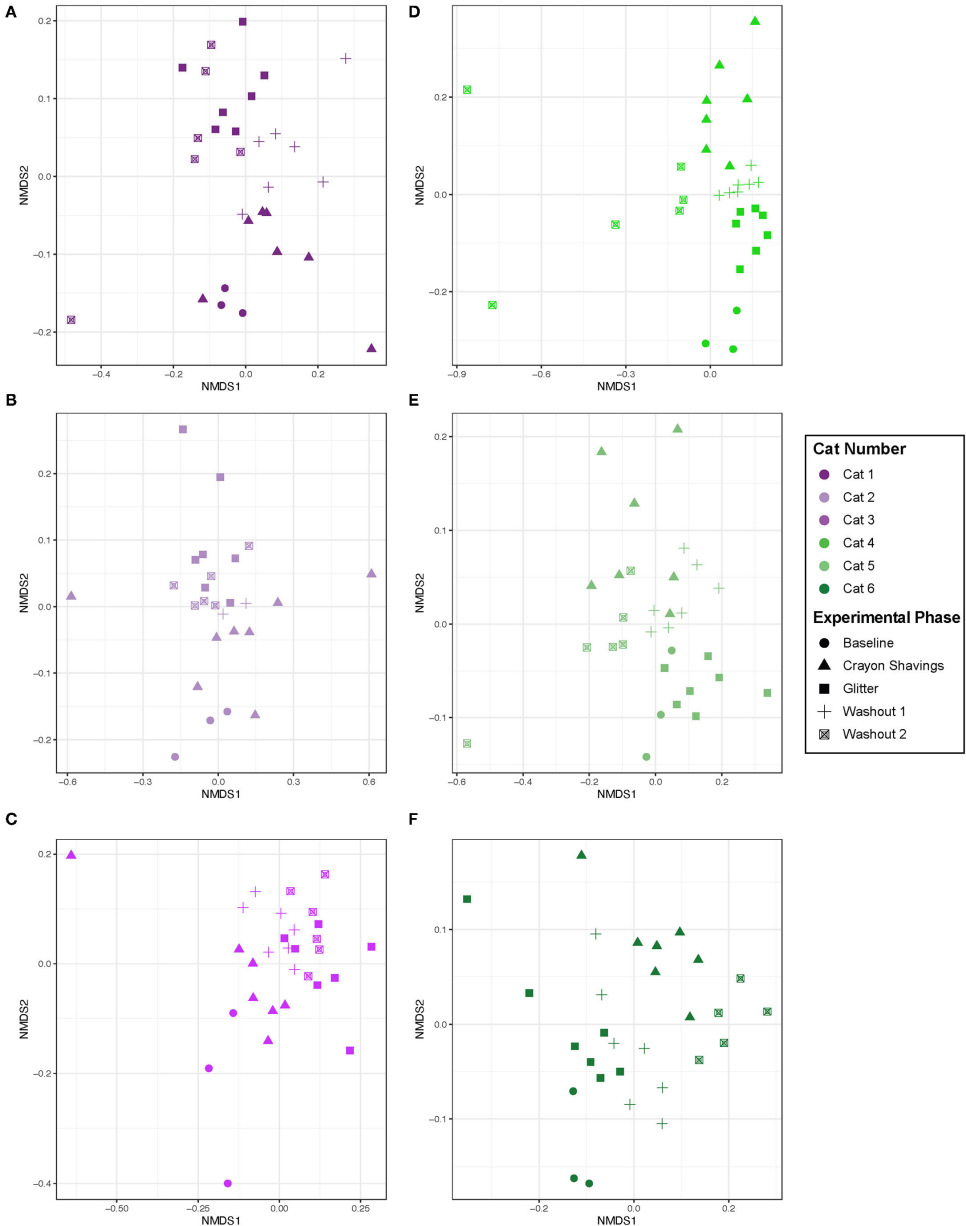
Microbial community structure was altered between experimental phases in an idiosyncratic pattern. NMDS ordination was calculated with Bray-Curtis dissimilarity algorithm on ASVs from fecal samples. Statistical significance of microbial community structure across experimental phase for each cat was determined with pairwise PERMANOVA analysis with a Benjamini-Hochberg *post-hoc* correction. Cat 6 had a single outlier (day 43 during W_2_ when hairball vomiting reported) that was removed from the NMDS plot to allow for better visualization of data. Each point represents an individual fecal sample from one cat. CG group is denoted in purple [Cats 1, 2, and 3; plots **(A–C)**, respectively]. GC group is denoted in green [Cats 4, 5, and 6; plots **(D–F)**, respectively]. Experimental phase is represented by a different symbol as denoted in the legend.

Given the cat-specific clustering observed, NMDS plots were generated for individual cats, where plot colors reflect the CG vs. GC marker order groups (Figure 5). Table 1 shows the PERMANOVA pairwise comparisons for individual cats. Across all comparisons of experimental changes, idiosyncratic changes to fecal microbial community structures were observed. Each cat responded differentially to treatment irrespective of the marker order such that there was no observable trend in microbial community changes across experimental phases for any of the pairwise comparisons assessed.

**Table 1:** PERMANOVA testing was calculated using Bray-Curtis distances.

**Cat Number**	**Cat 1 (R^2^ = 0.11)**	**Cat 2 (R^2^ = 0.12)**	**Cat 3 (R^2^ = 0.14)**	**Cat 4 (R^2^ = 0.25)**	**Cat 5 (R^2^ = 0.10)**	**Cat 6 (R^2^ = 0.24)**
Marker order	Marker _1_ = Crayon, Marker _2_ = Glitter			Marker _1_ = Glitter, Marker _2_ = Crayon		
Baseline vs. Marker 1	*p* = 0.1525	*p* = 0.2271	*p* = 0.4020	***p = 0.01250***	*p* = 0.1012	*p* = 0.08142
Baseline vs. Washout 1	*p* = 0.1400	*p* = 0.2271	*p* = 0.05800	***p = 0.01250***	*p* = 0.06428	*p* = 0.1477
Baseline vs. Marker 2	*p* = 0.05250	*p* = 0.2271	***p = 0.03000***	***p = 0.01250***	*p* = 0.2450	***p = 0.02750***
Baseline vs. Washout 2	*p* = 0.1400	*p* = 0.2271	*p* = 0.05800	*p* = 0.1120	*p* = 0.06428	*p* = 0.1477
Marker 1 vs. Washout 1	*p* = 0.7980	*p* = 0.4260	*p* = 0.0833	*p* = 0.07222	*p* = 0.06428	***p = 0.02750***
Marker 1 vs. Marker 2	***p = 0.02000***	*p* = 0.2271	***p = 0.03000***	***p = 0.007500***	***p = 0.01500***	***p = 0.02750***
Marker 1 vs. Washout 2	*p* = 0.05250	*p* = 0.2700	*p* = 0.06428	***p = 0.007500***	***p = 0.01500***	***p = 0.03000***
Washout 1 vs. Marker 2	*p* = 0.05250	*p* = 0.3377	***p = 0.03000***	***p = 0.01250***	*p* = 0.06428	*p* = 0.4240
Washout 1 vs. Washout 2	*p* = 0.1400	*p* = 0.2271	*p* = 0.06500	***p = 0.007500***	*p* = 0.05666	***p = 0.02750***
Marker 2 vs. Washout 2	*p* = 0.2055	*p* = 0.2271	*p* = 0.06428	***p = 0.007500***	*p* = 0.2450	***p = 0.03000***

R^2^ values refer to the percentage of variation in the model explained by the explanatory variable “experimental phase” (baseline, marker 1, washout 1, marker 2, washout 2). In each cell, values represented for each contrast are *p*-values, where *bold italics* indicate statistical significance. Significance was defined as *p* < 0.05 following Benjamini-Hochberg *post-hoc* correction.

### 3.3. Relative abundance and differential abundance at phylum and family level

**Figure 6:**
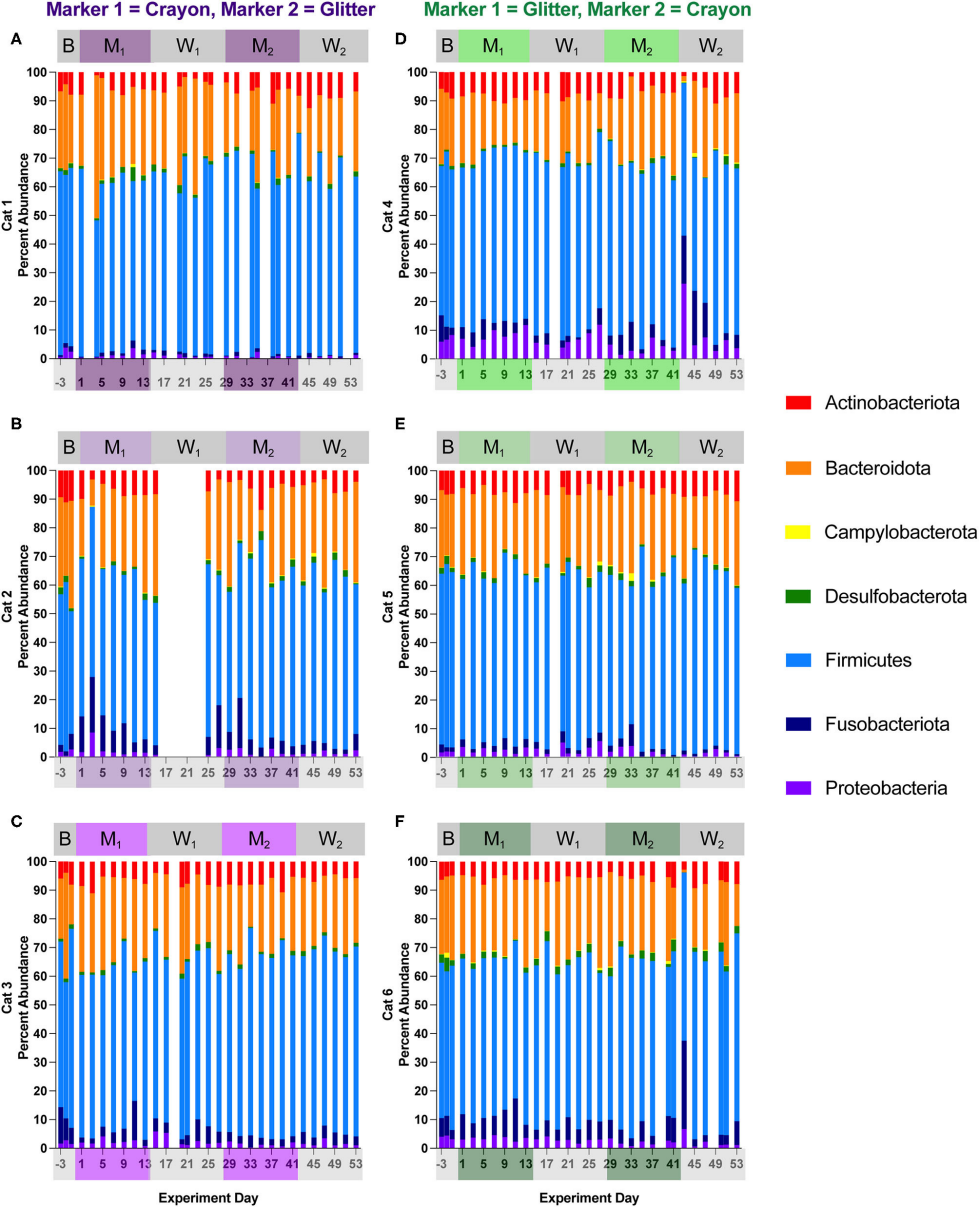
Cat-dependent shifts in phyla relative abundances occurred across all experimental phases. The composition of the fecal microbiota was visualized with bar plots of the phylum relative abundance for each cat (*n* = 3 per treatment group). CG group is denoted in purple [Cats 1, 2, and 3; bar plots **(A–C)**, respectively]. GC group is denoted in green [Cats 4, 5, and 6; bar plots **(D–F)**, respectively]. Days without data indicate that no feces were collected from overnight separation.

**Figure 7:**
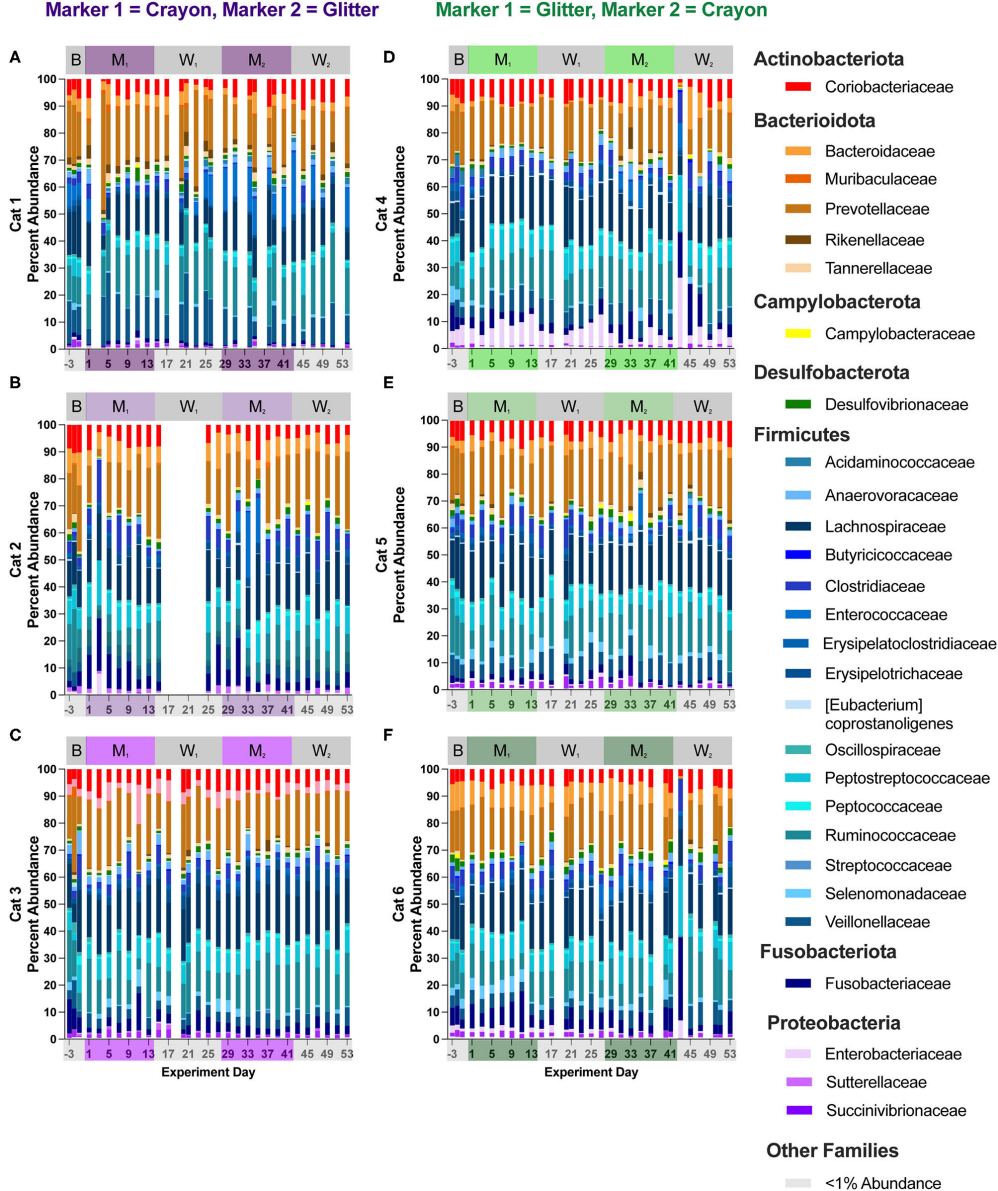
Cat-dependent shifts in family relative abundances occurred across all experimental phases. The composition of the fecal microbiota was visualized with bar plots of the family relative abundance for each cat (*n* = 3 per treatment group). CG group is denoted in purple [Cats 1, 2, and 3; bar plots **(A–C)**, respectively]. GC group is denoted in green [Cats 4, 5, and 6; bar plots **(D–F)**, respectively]. Days without data indicate that no feces were collected from overnight separation.

Figures 6, 7 show the relative abundances of microbial phyla and families, respectively for each cat and study day. Supplementary material 1 shows the percent relative abundances of all phylum and family ASV for each sample, and Supplementary Table 3 provides the log_2_ fold changes of the differentially abundant family members for each cat across different experimental phases. Eight phyla were identified across all cats and study phases. Among these phyla, *Firmicutes* was the most abundant, where its percent abundance ranged from 42.81 to 69.44% across cats during baseline, 44.76 to 65.61% during M_1_, 45.35 to 68.66% during W_1_, 48.17 to 72.38% M_2_, and 43.54 to 77.51% during W_2_. No significant differences in *Firmicutes* percent abundance were identified for any cat across any of the study phases. In two cats, phylum *Campylobacterota* was significantly decreased when comparing M_1_ vs. W_1_ (Cat 4, log_2_ fold decrease −1.54, *p* = 0.045), M_1_ vs. M_2_ (Cat 4, log_2_ fold decrease −2.34, *p* = 0.000033), Cat 5, log_2_ fold decrease −2.13, *p* = 0.0096), and baseline vs. M_2_ (Cat 4, log_2_ fold decrease −2.32, *p* = 0.0017). An acute decrease in *Bacteriodota* was seen during the Washout 2 phase between days 41–43 for Cats 4 and 6 (GC group). In Cat 4, between days 41–43, *Bacteriodota* decreased from 28.78 to 1.75% (mean 21.65 % ± 4.85% standard deviation, when averaging all other study days). In Cat 6, *Bacteriodota* decreased from 17.80 to 0.94% (mean 25.43 ± 4.49% standard deviation) over this timeframe. For both cats, following the acute drop on day 43, both *Bacteriodota* increased back toward their previous (i.e., prior to hairball vomiting episode) abundance levels within 2 days following the episode (Figures 6D–F). However, as these changes occurred within the middle of the W_2_, they were not detected as differential phyla abundances across any experimental phase comparisons (Supplementary material 2).

Fifty-three families were identified across all cats and study phases (Supplementary material 2). Changes in families grouped under the phylum *Firmicutes* represented the majority of differentially abundant families in five of the six cats (no differences observed in Cat 6). These differentially abundant families included *Acidaminococcaceae, Anaerovoracaceae, Clostridiaceae, Enterococcaceae, Erysipelatoclostridiaceae, Erysipelotrichaceae, Eubacteriaceae, Peptococcaceae*, and *Streptococcaceae*. Alterations to families associated with phylum *Actinobacteriota* were present in four cats across all experimental phases and included *Atopobiaeae, Eggerthellaceae*, and *Bifidobacteriaceae*. For *Firmicutes*-associated families, there were no discernible pattens of increases or decreases for any cat and across any pairs of experimental phases. For the *Actinobacteriota*-associated families, three cats (Cat 3, Cat 4, Cat 6) had significant alterations to family *Bifidobacteriaceae* across experimental phases. In Cat 3, this included a log_2_ fold decrease of −7.20 when comparing baseline vs. M_1_ (*p* = 0.00021) and a log_2_ fold decrease of −7.05 when comparing baseline to M_2_ (*p* = 0.00034). In Cat 4, increased *Bifidobacteriaceae* was observed when comparing W_1_ to M_2_ (log_2_ fold increase 2.32, p = 0.016). For Cat 6, increased *Bifidobacteriaceae* was seen when comparing W_1_ to M_2_ (log_2_ fold increase 2.12, *p* = 0.0088), when comparing M_1_ to M_2_ (log_2_ fold increase 2.22, *p* = 0.0040), and for baseline vs. M_2_ (log_2_ fold increase 2.46, *p* = 0.030).

In Cat 4 and Cat 6 changes in family abundances were observed between days 41 and 43 (acute hairball vomiting episode). For Cat 4, when comparing day 41 vs. day 43, these changes included increases in *Peptostreptococcaceae* from 5.49 to 20.14%, (mean of 16.48 ± 3.91% standard deviation, when averaging all other study days), increased *Enterobacteriaceae* from 1.57 to 25.91% (mean 5.43 ± 2.87% standard deviation), and decreased *Prevotellaceae* from 23.71 to 0.61% (mean 16.48 ± 3.91% standard deviation). In Cat 6, when comparing days 41 and 43, *Fusobacteriaceae* increased from 8.64 to 30.84% (6.04% mean ± 2.78% standard deviation when averaging all other study days), *Peptostreptococcaceae* increased from 8.86 to 25.65% (mean 7.18 ± 1.82% standard deviation), and *Prevotellaceae* decreased from 10.89 to 0.44% (mean 16.42% ± 3.32% standard deviation). Similar to the trends observed at the phylum level, family relative abundances returned to their previous levels by day 45 through the end of the study, and no changes in family differential abundances were impacted by this acute shift, as it occurred in the middle of the second washout phase, at which time cats were no longer consumer either marker (Figures 7D, F, Supplementary material 2).

## 4. Discussion

The objective of this study was to determine whether orally administered fecal identification markers, glitter and crayon shavings, altered the fecal microbial community structure in healthy adult domestic cats. While body weights, fecal scores, and health status remained consistent for all cats throughout the study, cats demonstrated idiosyncratic fecal microbiota shifts during oral administration of both glitter and crayon shavings that were independent of the marker treatment order. Consequently, downstream analysis focused on differences in the fecal microbial community composition between experimental phases for each individual cat, where each cat served as its own internal comparison. Similar phenomena have been demonstrated in several other feline studies including across cats of different ages and intestinal regions, during antibiotic use, in the presence of a synbiotic, and irrespective of sampling technique (25–28).

Fecal score and body weight were assessed throughout the study given their known impacts on the gut microbiota (29, 30). Weight changes were minimal for all cats throughout the study, as they were fed and consumed their maintenance caloric needs, suggesting that weight fluctuations were not a primary driver of observed microbial community alterations observed during this study. Minor but abrupt changes in fecal consistency have been associated with microbiota shifts in cats (29). Additionally, stool consistency is an important aspect of feline health, and some owners and practitioners may be hesitant to use fecal identification markers if they inherently cause diarrhea. Given that fecal scores were elevated at baseline for all cats (mean score of 4.5 for group CG, mean score of 6 for group GC), the effects of glitter or crayon shavings alone on fecal score cannot be inferred. Fecal scores remained consistently high throughout the 56-day study and did not increase or decrease significantly between CG and GC groups during any of the experimental phases (Figure 2B). These findings suggest that the high fecal scores observed for all cats herein may be related to the transition to an exclusively canned diet three weeks prior to study initiation vs. secondary to addition of fecal markers (glitter or crayon shavings).

Diet alterations, including fiber content, are known to modulate bowel movement frequency and fecal consistency in cats (31, 32). Consequently, modulations to dietary fiber offer one explanation for the higher fecal scores identified in this study. Fiber content decreased from 0.72 grams/100 kcal in the dry food formulation to 0.15 grams/100 kcal in the canned diet (Supplementary Table 1). One previous study observed that cats had higher fecal scores when transitioning onto pectin or fructooligosaccharide-containing diets, but otherwise no other adverse health concerns were reported (25). Additional studies have reported decreased stool consistency (i.e., less formed and with higher moisture) when healthy cats were supplemented a variety of fibers ranging from 3 to 11% on an as-fed basis (33, 34), and it is possible that a similar response occurred in this study. Within this feline clinically healthy study population, there is no suspicion for a food-responsive and/or fiber-responsive enteropathy, as fecal scores normalized after this study when cats were transitioned back to their original low fiber dry diet. This provides evidence that increased fecal consistency was largely impacted by dietary formulation change in this population of cats.

Alpha diversity, which measures the microbial community diversity of an individual fecal sample, has been previously noted to fluctuate with acute and chronic changes to the feline gastrointestinal environment including with antibiotic use and in chronic enteropathy (7, 27). Alpha diversity did not vary when comparing the mean scores of CG group to GC group (Figure 3) across any experimental phase. These findings support that the overall diversity within individual fecal samples was not significantly affected by glitter or crayon shaving administration. When assessing inter-sample diversity (beta diversity), fecal microbial communities were most similar within individual cats such that each cat had a distinct fecal microbiome composition (*p* < 0.0001 for all cat comparisons) and no differences across experimental phases or between groups CG and GC were observed (Figure 4).

Given the idiosyncratic response observed herein, each cat was assessed individually for fecal microbial community changes (Figure 5). One cat (Cat 2) demonstrated no significant alterations in the fecal microbial community structure throughout the study. However, in five of the six cats, significant differences in the fecal microbial community structure were observed across different experimental phases, but they were not consistent between cats (Table 1). Both temporal and fecal marker changes accounted for these fecal microbiota community shifts. Cat 1, Cat 5, and Cat 6 demonstrate temporal, but not marker, dependent shifts in the fecal microbiota over the course of this study. For these cats, no significant shifts to the fecal microbiota are seen when comparing M_1_ to W_1_ and baseline, nor are shifts seen when comparing M_2_ to both W_2_ and W_1_. However, there are significant shifts in the fecal microbial community composition when comparing non-consecutive experimental phases, such as M_1_ vs. M_2_. Families that contributed to these temporal shifts included increased *Clostridiaceae* in Cat 1 (3.07 log_2_ fold increase, *p* = 0.0001) and Cat 5 (1.46 log_2_ fold increase, *p* = 0.026) and decreased *Bifidobacteriaceae* in Cat 6 (−2.22 log_2_ fold decrease, *p* = 0.0040; Supplementary Table 2). These families are naturally abundant in the healthy adult feline fecal microbiota (35). Extrinsic factors (i.e., weight checks, physical examinations, and changes to daily personnel handling each cat) and/or normal physiologic variation modulating the gut microbiota may be driving these temporal shifts. Acute environmental stressors have been shown to modulate the gut microbiota in people and mice (36–38). However, no studies in cats have explored the impact of acute environmental stressors on the gut microbiota. Additionally, normal physiologic variation in the feline gut microbiota is ill defined.

In contrast to time-dependent modulations, fecal markers modulated the microbiota in Cat 3 and Cat 4. For these cats, modulations to the fecal microbiota were seen across the experimental phases that were directly preceding or following administration of fecal markers. In Cat 3, significant differences between the W_1_ and subsequent M_2_ (glitter administration) fecal microbial communities support that the addition of glitter modulated the fecal microbiota. In Cat 4, significant differences between baseline and M_1_ (glitter administration) and between W_1_ and M_2_ (crayon administration) indicate that both fecal identification markers modulated the fecal microbiota. The decreased *Acidaminococcaceae* (−5.91 log_2_ fold decrease, *p* = 0.014) and *Streptococcaceae* (−6.21 log_2_ fold decrease, *p* = 0.030) in Cat 3 and decreased *Eggerthellaceae* (−1.97 log_2_ fold decrease, *p* = 0.0000049) and *Anaerovoracaceae* (−2.65 log_2_ fold decrease, *p* = 0.016) in Cat 4 can be explored further as markers of glitter-mediated fecal microbiota modulations in cats (Supplementary Table 2). Similarly, the impact of oral crayon shavings on increased fecal *Bifidobacteriaceae* (log_2_ fold increase 2.32, p = 0.016, Cat 4) can be assessed, given the roles that *Bifidobacteria* play in digestion, host colonic health, and immune function (Supplementary Table 2) (39). Furthermore, the lack of differences between the M_2_ and W_2_ fecal communities for Cat 3 demonstrates that these fecal marker-driven changes persisted through the entire 14-day washout period. Therefore, a longer washout time may therefore be needed if using these markers in conjunction with fecal microbiota analyses. This was challenging to assess in Cat 6, who experienced hairball vomiting during W_2_.

Alongside temporal and fecal identification marker-driven impacts on the feline fecal microbiota, the acute changes to the fecal microbiota composition for Cats 4 and 6, occurring between days 41–43, coincided with hairball vomiting. There are no studies assessing fecal microbiota changes in cats with transient acute vomiting secondary to hairballs. However, other acute gastrointestinal disturbances, including diarrhea, are known to alter the fecal microbial community structure in cats, where increases in phyla *Firmicutes* and *Proteobacteria* have been observed (29). Although these taxa were not impacted by vomiting in this study, the acute decrease in phylum *Bacteriodota* and family *Prevotellaceae*, as well as increases in the families *Enterobacteriaceae, Peptostreptococcaceae*, and *Fusobacteriaceae* can be explored in future studies as microbiota signatures associated with feline acute vomiting. These results additionally emphasize the impact that sampling frequency (i.e., daily sampling vs. weekly sampling) had on the potential outcomes of this study. For example, if sampling had only been performed on one day of each experimental phase, the acute microbiota change associated with vomiting may have been missed, or if included, it may have skewed interpretation of fecal compositional changes over time. Daily fluctuations to the healthy microbiota, which are known to occur people, are not yet established for domestic cats and are likely additional contributors to microbiota abundance changes identified across experimental phases (27, 40).

Limitations of this study include a small sample size (*n* = 6 cats) which may not have been powered enough to overcome individual cat differences to identify changes in the microbiota in response to each fecal marker and over time. There is no standard for calculating power for studies with microbiome and metagenome sequence-based analyses, as calculating effect sizes from a compositional dataset may not accurately reflect the true magnitude of difference between treatments and/or be clinically relevant (41–43). Approximately 10–25% of the variation in microbial community structure between samples was attributed to differences across experimental phases, supporting that there is a contribution of both experimental phase and potentially marker order on the fecal microbiota. The impact of time on the fecal microbiota may additionally serve as a confounding variable here, including shifts across experimental phases secondary to marker order. However, given that only one of the six cats examined (Cat 4, Table 1) showed a difference between the baseline and washout 1 timepoints, and no cats showed a difference in community structure when comparing baseline to washout 2, time is not anticipated to be a driving factor in the differences reported herein. In light of these confounding variables, it is unclear whether alterations in microbial phyla and families that were observed herein were exclusively related to administration of glitter and crayon fecal markers. Although this study did not identify any adverse clinical effects in cats that were directly attributable to glitter or crayon shavings, the long-term effects of these markers on clinical health are unknown. However, long-term clinical adverse effects are not anticipated given the overall lack of reported adverse events across other studies assessing fecal marker use in client-owned cats (11–13).

Furthermore, the impact of sampling frequency on microbial community changes is not established. Differences across fecal markers for each cat may reflect, in part, normal daily fluctuations in microbial abundance vs. changes driven by glitter or crayon shavings. Establishing this effect of sampling frequency may additionally be important before using fecal markers in future studies with microbiota endpoints. The impact of glitter and crayon shavings on fecal samples used for calculating the feline dysbiosis index, which uses qPCR to estimate the abundance of several key members within the fecal microbiota, should additionally be assessed, as this index quantifies the abundance of Bifidobacteria and other genera associated with the differentially abundant phyla and families herein (44). Although dysbiosis is defined with this index for cats with chronic enteropathies (44), the index has not been validated for other populations of cats, including for healthy cats receiving fecal identification markers nor has it been examined for changes over time (e.g., days to weeks) in a population of domestic cats.

Although the impacts of glitter and crayon shavings on the feline fecal microbiota merit further investigation, these markers continue to serve as useful tools for distinguishing feces among individual cats. In multi-cat households, laboratory facilities, and shelters, glitter and crayon shavings can function as an affordable, well-tolerated oral regimen which disappear from the feces within days of discontinuing them. Clinical scenarios where oral administration of glitter and/or crayon shavings may be useful include to differentiate between cats for routine diagnostics such as fecal floats, to identify cats with diarrhea, to evaluate litterbox preferences, and to assess for inappropriate defecation outside of the litterbox.

## 5. Conclusions

Oral administration of crayon shavings and glitter are well tolerated in cats and easily observed in the feces, supporting their use in clinical practice. However, crayon shavings and glitter showed statistically significant variations in individual cats' fecal microbial community structures, including those measured on the feline dysbiosis index, and therefore should be used with caution when diagnostic and/or research include microbiota endpoints until the impact of sampling frequency and other factors, including diet and stress on the microbiota, can be better elucidated. Research investigating fecal sampling frequency and feline daily microbial community dynamics in both research facilities and in home settings is warranted and could expand the utility of these fecal identification markers.

## Data availability statement

The datasets presented in this study can be found in online repositories. The names of the repository/repositories and accession number(s) can be found in the article/Supplementary material.

## Ethics statement

The animal study was reviewed and approved by the Ohio State University Institutional Animal Care and Use Committee (2017A00000093-R1).

## Author contributions

Conceptualization: JW, AR, JH, and VP. Funding acquisition: JW, AR, JH, and JQ. Project administration: JW and AR. Sample collection: HK, MS, and AR. Data curation: NN and HK. Microbiome analysis, data analysis, and visualization: NN and JW. Writing—original draft: NN and AW. Writing—review and editing: JW, NN, AW, AR, VP, and JQ. All authors contributed to this article and approved the submitted version.
