# Supplementary material for: Fecal identification markers impact the feline fecal microbiota
Source: Front Vet Sci. 2023 Feb 8;10:1039931. doi: 10.3389/fvets.2023.1039931 (PMC9946173; doi:10.3389/fvets.2023.1039931)
Supplement: Supplementary file 1 [file Table_1.DOCX]

| **Supplementary Table 1. Nutrient Profiles of Pre-Study and Study Diets** | | |
| --- | --- | --- |
| **Nutrient (g/100 kcal)** | **Cincinnati Lab Supply Inc.**  **Laboratory Feline Diet 5003** | **Purina Pro Plan Adult Chicken & Rice Entrée in Gravy** |
| Crude Protein | 9.97 | 15.42 |
| Crude Fat | 3.76 | 3.61 |
| Crude Fiber | 0.72 | 0.15 |
| Metabolizable Energy | 3.06 kcal/gram | 0.83 kcal/gram |
| **Ingredients** | Dehulled soybean meal, ground corn, chicken meal, corn gluten meal, rice flour, porcine animal fat, poultry fat, brewers dried yeast, soybean oil, wheat germ, dried whey, beet pulp, fish meal, amino acids, vitamins and minerals | Water sufficient for processing, chicken, wheat gluten, liver, meat by-products, rice, corn starch-modified, soy protein concentrate, amino acids, vitamins and minerals |
