# Supplementary material for: Fecal identification markers impact the feline fecal microbiota
Source: Front Vet Sci. 2023 Feb 8;10:1039931. doi: 10.3389/fvets.2023.1039931 (PMC9946173; doi:10.3389/fvets.2023.1039931)
Supplement: Supplementary file 2 [file Table_2.DOCX]

| **Supplementary Table 2. Purina Fecal Scoring Chart Used to Score Daily Fecal Samples** | |
| --- | --- |
| **Score** | **Characteristics** |
| **1** | - Very hard and dry - Often expelled as individual pellets - Requires much effort to expel from body - Leaves no residue on ground when picked up |
| **2** | - Firm, but not hard, pliable - Segmented in appearance - Little or no residue on ground when picked up |
| **3** | - Log shaped, moist surface - Little or no visible segmentation - Leaves residue on ground, but holds form when picked up |
| **4** | - Very moist and soggy - Log shaped - Leaves residue on ground and loses form when picked up |
| **5** | - Very moist but has a distinct shape - Present in piles rather than logs - Leaves residue on ground and loses form when picked up |
| **6** | - Has texture, but no defined shape - Present as piles or spots - Leaves residue on ground when picked up |
| **7** | - Watery - No texture - Present in flat puddles |
| **Reference**: https://www.purinainstitute.com/centresquare/nutritional-and-clinical-assessment/purina-fecal-scoring-chart | |
