# Supplementary material for: Fecal identification markers impact the feline fecal microbiota
Source: Front Vet Sci. 2023 Feb 8;10:1039931. doi: 10.3389/fvets.2023.1039931 (PMC9946173; doi:10.3389/fvets.2023.1039931)
Supplement: Supplementary file 3 [file Table_3.DOCX]

| **Supplementary Table 3. Differentially abundant families across experimental phases for individual cats** | | | | | | | | | | | | |  |
| --- | --- | --- | --- | --- | --- | --- | --- | --- | --- | --- | --- | --- | --- |
| **Phylum** | **Family** | **Baseline / Marker 1  Log_2_ Fold Change** | | | | | | | | | | |  |
|  |  | **Marker _1_ = Crayon, Marker _2_ = Glitter** | | | | | **Marker _1_ = Glitter, Marker _2_ = Crayon** | | | | | |  |
|  |  | **C1** | **C2** | **C3** | | | **C4** | **C5** | | **C6** | | |  |
| Actinobacteriota | Atopobiaceae | **↓** -0.60 ± 0.31 *(0.23)* | **↓** -0.06 ± 0.69 *(1.00)* | **↓ -3.32 ± 0.85 *(0.0015)*** | | | **↓** -1.09 ± 1.29 *(0.82)* | **↑** 0.10 ± 0.37 *(1.00)* | | **↑** 0.24 ± 0.71 *(1.00)* | | |  |
|  | Bifidobacteriaceae | **↓** -0.55 ± 0.64 *(0.90)* | **↑** 1.27 ± 0.84 *(0.70)* | **↓ -7.20 ± 1.62 *(0.00021)*** | | | **↓** -1.94 ± 0.93 *(0.23)* | **↓** -0.74 ± 0.55 *(0.86)* | | **↑** 0.23 ± 0.71 *(1.00)* | | |  |
|  | Eggerthellaceae | **↓** -0.018 ± 0.33 *(1.00)* | **↓** -0.91 ± 0.46 *(0.37)* | **↑** 0.26 ± 0.27 *(1.00)* | | | **↓ -1.97 ± 0.36 *(4.90 E-6)*** | **↑** 0.11 ± 0.21 *(1.00)* | | **↑** 0.23 ± 0.29 *(1.00)* | | |  |
| Bacteroidota | Muribaculaceae | **↑ 1.34** ± **0.37 (*0.0068*)** | **↑** 0.11 ± 0.58 *(1.00)* | **↑** 1.13 ± 0.49 *(0.17)* | | | **↑** 0.62 ± 0.50 *(0.62)* | **↑** 0.65 ± 0.40 *(0.86)* | | **↓** -0.10 ± 0.46 *(1.00)* | | |  |
| Firmicutes | Acidaminococcaceae | **↓ -1.68 ± 0.59 *(0.047)*** | - | **↑** 0.71 ± 2.32 *(1.00)* | | | **↑** 2.21 ± 2.49 *(0.82)* | **↑ 4.41 ± 0.94 *(0.00016)*** | | - | | |  |
|  | Anaerovoracaceae | **↓** -0.26 ± 0.38 *(0.99)* | **↓** -1.04 ± 0.51 *(0.37)* | **↑** 0.80 ± 0.54 *(0.62)* | | | **↓ -2.65 ± 0.77 *(0.016)*** | **↑** 0.40 ± 0.42 *(0.95)* | | **↓** -0.080 ± 0.43 *(1.00)* | | |  |
|  | Clostridiaceae | **↑ 3.07 ± 0.64 *(0.00010)*** | **↓** -1.72 ± 0.73 *(0.31)* | **↑** 0.013 ± 0.63 *(1.00)* | | | **↑** 0.064 ± 0.70 *(1.00)* | **↓** -0.87 ± 0.64 *(0.86)* | | - | | |  |
|  | Enterococcaceae | **↓** -3.55 ± 1.62 *(0.15)* | **↓** -1.91 ± 1.83 *(0.88)* | **↓ -21.90 ± 2.08**  ***(3.52 E-24)*** | | | **↓** -2.01 ± 1.88 *(0.73)* | **↑** 1.15 ± 1.88 *(0.99)* | | **↑** 1.25 ± 1.48 *(1.00)* | | |  |
|  | Erysipelatoclostridiaceae | **↑ 1.76 ± 0.56 *(0.021)*** | **↓** -0.73 ± 0.76 *(0.88)* | **↓ -1.69 ± 0.47 *(0.0050)*** | | | **↑** 2.34 ± 0.92 *(0.13)* | **↓** -1.04 ± 0.67 *(0.86)* | | **↓** -0.79 ± 0.80 *(1.00)* | | |  |
|  | Erysipelotrichaceae | **↓** -0.58 ± 0.38 *(0.37)* | **↓ -2.87 ± 0.37 *(1.38 E-12)*** | **↓** -0.12 ± 0.34 *(1.00)* | | | **↑** 1.03 ± 0.64 *(0.42)* | **↓** -1.24 ± 0.44 *(0.13)* | | **↓** -0.89 ± 0.76 *(1.00)* | | |  |
|  | Eubacteriaceae | **↓ -5.72 ± 1.44 *(0.0019)*** | - | - | | | - | - | | - | | |  |
| **Phylum** | **Family** | **Baseline / Washout 1 Log_2_ Fold Change** | | | | | | | | | | |  |
|  |  | **Marker _1_ = Crayon, Marker _2_ = Glitter** | | | | **Marker _1_ = Glitter, Marker _2_ = Crayon** | | | | | | |  |
|  |  | **C1** | **C2** | | **C3** | **C4** | | | **C5** | | **C6** | |  |
| Actinobacteriota | Atopobiaceae | **↑** 0.97 ± 0.39 *(0.25)* | **↑** 1.08 ± 0.99 *(1.00)* | | **↓ -2.81 ± 0.85 *(0.010)*** | **↑** 1.14 ± 1.34 *(0.66)* | | | **↓** -0.033 ± 0.37 *(1.00)* | | **↓** -0.31 ± 0.71 *(1.00)* | |  |
|  | Bifidobacteriaceae | **↓** -1.10 ± 0.80 *(0.76)* | **↑** 1.73 ± 1.14 *(0.78)* | | **↓ -5.74 ± 1.62 *(0.0093)*** | **↓** -2.07 ± 0.93 *(0.097)* | | | **↓** -1.10 ± 0.55 *(0.61)* | | **↑** 0.34 ± 0.71 *(1.00)* | |  |
|  | Eggerthellaceae | **↑** 0.34 ± 0.42 *(1.00)* | **↓** -0.83 ± 0.62 *(0.84)* | | **↑** 0.31 ± 0.27 *(0.91)* | **↓ -2.13 ± 0.36 *(3.14 E-7)*** | | | **↑** 0.57 ± 0.22 *(0.25)* | | **↓** -0.053 ± 0.29 *(1.00)* | |  |
| Firmicutes | Acidaminococcaceae | **↓ -4.41 ± 0.77 *(7.80 E-7)*** | - | | **↑** 0.27 ± 2.32 *(1.00)* | **↑ 11.70 ± 2.58 *(0.00011)*** | | | **↑ 4.91 ± 0.95 *(1.47 E-5)*** | | - | |  |
|  | Anaerovoracaceae | **↑** 0.081 ± 0.49 *(1.00)* | **↓** -1.64 ± 0.69 *(0.44)* | | **↑** 0.76 ± 0.54 *(0.73)* | **↓** **-3.24 ± 0.77 *(0.00039)*** | | | **↑** 0.95 ± 0.42 *(0.42)* | | **↓** -0.43 ± 0.43 *(1.00)* | |  |
|  | Enterococcaceae | **↑** 0.56 ± 2.09 *(1.00)* | **↓** -1.30 ± 2.47 *(1.00)* | | **↓ -21.35 ± 2.08 *(5.71 E-23)*** | **↓** -2.88 ± 1.88 *(0.31)* | | | **↑** 1.85 ± 1.88 *(0.98)* | | **↓** -0.20 ± 1.48 *(1.00)* | |  |
|  | Erysipelatoclostridiaceae | **↑** 0.77 ± 0.71 *(0.83)* | **↑** 0.84 ± 1.03 *(1.00)* | | **↓ -1.60 ± 0.47 *(0.010)*** | **↑** 1.71 ± 0.92 *(0.19)* | | | **↓** -0.02 ± 0.67 *(1.00)* | | **↓** -0.78 ± 0.80 *(1.00)* | |  |
|  | Erysipelotrichaceae | **↑** 0.94 ± 0.49 *(0.50)* | **↓ -2.68 ± 0.50 *(6.56 E-6)*** | | **↓** -0.71 ± 0.34 *(0.26)* | **↑** 0.16 ± 0.64 *(0.86)* | | | **↓** -0.25 ± 0.44 *(1.00)* | | **↓** -1.32 ± 0.76 *(1.00)* | |  |
|  | Lachnospiraceae | **↑** 0.37 ± 0.24 *(0.73)* | **↓** -0.15 ± 0.46 *(1.00)* | | **↓** -0.18 ± 0.24 *(1.00)* | **↓ -0.71 ± 0.23 *(0.011)*** | | | **↓** -0.0084 ± 0.23 *(1.00)* | | - | |  |
|  | Oscillospiraceae | **↓** -0.014 ± 0.42 *(1.00)* | **↑** 0.13 ± 0.70 *(1.00)* | | **↑ 1.48 ± 0.43 *(0.0093)*** | **↑** 0.96 ± 0.40 *(0.074)* | | | **↑** 0.33 ± 0.46 *(1.00)* | | **↑** 0.31 ± 0.39 *(1.00)* | |  |
|  | Selenomonadaceae | **↑** 0.45 ± 0.72 *(1.00)* | **↑** 0.62 ± 1.08 *(1.00)* | | **↓** -0.43 ± 1.02 *(1.00)* | **↑ 3.07 ± 0.94 *(0.010)*** | | | **↓** -1.41 ± 0.77 *(0.61)* | | **↑** 0.67 ± 0.69 *(1.00)* | |  |
|  | Unclassified ^a.^ | **↓** -1.29 ± 0.62 *(0.45)* | **↓** -0.28 ± 0.89 *(1.00)* | | **↑** 0.44 ± 0.39 *(0.91)* | **↑ 1.71 ± 0.55 *(0.011)*** | | | **↓** -0.21 ± 0.45 *(1.00)* | | **↑** 0.64 ± 0.48 *(1.00)* | |  |
| **Phylum** | **Family** | **Baseline / Marker 2 Log_2_ Fold Change** | | | | | | | | | | |  |
|  |  | **Marker _1_ = Crayon, Marker _2_ = Glitter** | | | | | **Marker _1_ = Glitter, Marker _2_ = Crayon** | | | | | |  |
|  |  | **C1** | **C2** | **C3** | | | **C4** | **C5** | | **C6** | | |  |
| Actinobacteriota | Atopobiaceae | ↑ 0.048 ± 0.39 *(1.00)* | ↑ 0.15 ± 0.70 *(1.00)* | **↓ -3.27 ± 0.85 *(0.0020)*** | | | ↑ 2.18 ± 1.41 *(0.40)* | ↓ -0.33 ± 0.37 *(1.00)* | | ↑ 0.87 ± 0.72 *(1.00)* | | |  |
|  | Bifidobacteriaceae | ↓ -0.39 ± 0.81 *(1.00)* | ↑ 2.26 ± 0.86 *(0.069)* | **↓ -7.05 ± 1.62 *(0.00033)*** | | | ↑ 0.24 ± 0.94 *(0.95)* | ↓ -0.84 ± 0.55 *(0.76)* | | **↑ 2.46 ± 0.72  *(0.030)*** | | |  |
|  | Eggerthellaceae | ↑ 0.21 ± 0.42 *(1.00)* | **↓ -1.39 ± 0.46 *(0.028)*** | ↑ 0.24 ± 0.27 *(0.84)* | | | **↓ -1.43 ± 0.37 *(0.0013)*** | ↑ 0.44 ± 0.22 *(0.38)* | | ↑ 0.14 ± 0.29 *(1.00)* | | |  |
| Bacteroidota | Rikenellaceae | ↑ 0.099 ± 0.53 *(1.00)* | ↑ 0.88 ± 0.99 *(1.00)* | ↓ -1.62 ± 0.99 *(0.36)* | | | **↓ -4.11 ± 1.00 *(0.0011)*** | ↓ -0.76 ± 0.89 *(1.00)* | | ↓ -1.12 ± 0.96 *(1.00)* | | |  |
| Firmicutes | Acidaminococcaceae | **↓** **-4.37 ± 0.77 *(1.07 E-6)*** | - | **↓ -5.64 ± 2.19** *(0.059)* | | | **↑** **11.68 ± 2.59 *(0.00032)*** | ↑ 2.46 ± 0.93 *(0.13)* | | - | | |  |
|  | Anaerovoracaceae | ↓ -0.40 ± 0.49 *(1.00)* | **↓ -1.63 ± 0.52 *(0.022)*** | ↑ 0.44 ± 0.54 *(0.84)* | | | **↓ -3.09 ± 0.77 *(0.0011)*** | ↑ 0.39 ± 0.42 *(1.00)* | | ↓ -0.47 ± 0.43 *(1.00)* | | |  |
|  | Clostridiaceae | **↑ 3.15 ± 0.83 *(0.0027)*** | ↓ -0.81 ± 0.74 *(0.95)* | ↑ 0.66 ± 0.63 *(0.77)* | | | ↑ 1.25 ± 0.70 *(0.28)* | ↑ 0.58 ± 0.64 *(1.00)* | | - | | |  |
|  | Enterococcaceae | ↓ -0.97 ± 2.09 *(1.00)* | **↓ -7.74 ± 1.87 *(0.00086)*** | **↓ -24.18 ± 2.08 *(1.73 E-29)*** | | | **↓ -5.75 ± 1.88 *(0.022)*** | ↓ -4.87 ± 1.87 *(0.13)* | | ↓ -1.33 ± 1.48 *(1.00)* | | |  |
|  | Erysipelatoclostridiaceae | **↑ 2.72 ± 0.72 *(0.0027)*** | ↓ 0.32 ± 0.77 *(1.00)* | **↓ -1.49 ± 0.47 *(0.022)*** | | | **↑ 2.77 ± 0.92 *(0.022)*** | ↑ 1.25 ± 0.67 *(0.43)* | | ↓ -0.46 ± 0.80 *(1.00)* | | |  |
|  | Erysipelotrichaceae | ↑ 0.30 ± 0.49 *(1.00)* | **↓ -3.36 ± 0.38 *(1.38 E-16)*** | ↓ -0.80 ± 0.34 *(0.078)* | | | ↑ 0.25 ± 0.64 *(0.95)* | ↓ -0.059 ± 0.44 *(1.00)* | | ↓ -1.32 ± 0.76 *(0.84)* | | |  |
|  | Peptococcaceae | ↓ -0.48 ± 0.36 *(0.83)* | **↓ -1.85 ± 0.54 *(0.010)*** | ↓ -0.17 ± 0.49 *(1.00)* | | | ↑ 0.15 ± 0.45 *(0.95)* | ↑ 0.15 ± 0.34 *(1.00)* | | ↓ -0.82 ± 0.29 *(0.10)* | | |  |
|  | Streptococcaceae | ↑ 1.97 ± 4.57 *(1.00)* | ↓ -4.03 ± 2.58 (*0.64)* | ↓ -6.72 ± 2.52 *(0.059)* | | | ↓ -3.40 ± 3.03 *(0.58)* | **↓ -7.58 ± 2.27 *(0.046)*** | | ↓ -4.16 ± 2.53 *(0.84)* | | |  |
| **Phylum** | **Family** | **Baseline / Washout 2 Log_2_ Fold Change** | | | | | | | | | | |  |
|  |  | **Marker _1_ = Crayon, Marker _2_ = Glitter** | | | | | **Marker _1_ = Glitter, Marker _2_ = Crayon** | | | | | |  |
|  |  | **C1** | **C2** | **C3** | | | **C4** | **C5** | | **C6** | | |  |
| Actinobacteriota | Bifidobacteriaceae | ↓ -1.23 ± 0.82 *(0.69)* | ↑ 2.54 ± 0.88 *(0.098)* | ↓ -4.62 ± 1.66 *(0.055)* | | | **↑ 2.79 ± 1.00 *(0.029)*** | ↓ -0.58 ± 0.56 *(1.00)* | | ↑ 1.81 ± 0.74 *(0.092)* | | |  |
|  | Coriobacteriaceae | ↓ -0.63 ± 0.41 *(0.69)* | ↓ 0.91 ± 0.45 *(0.23)* | ↑ 0.022 ± 0.36 *(1.00)* | | | ↑ 0.088 ± 0.38 *(1.00)* | ↓ -0.36 ± 0.29 *(1.00)* | | **↓ -1.05 ± 0.34 *(0.027)*** | | |  |
|  | Eggerthellaceae | ↓ -0.38 ± 0.43 *(0.90)* | ↓ -0.98 ± 0.47 *(0.23)* | ↑ 0.12 ± 0.28 *(1.00)* | | | **↓ -1.91 ± 0.37 *(1.20 E-5)*** | ↑ 0.27 ± 0.22 *(1.00)* | | ↓ -0.48 ± 0.30 *(0.35)* | | |  |
| Bacteroidota | Bacteroidaceae | ↑ 0.39 ± 0.39 *(0.90)* | ↑ 0.52 ± 0.46 *(0.91)* | ↑ 0.035 ± 0.57 *(1.00)* | | | ↓ -1.09 ± 0.68 *(0.29)* | ↓ -0.17 ± 0.57 *(1.00)* | | **↑ 1.20 ± 0.40 *(0.027)*** | | |  |
|  | Rikenellaceae | ↓ -0.14 ± 0.55 *(1.00)* | ↑ 2.59 ± 1.03 *(0.20)* | ↓ -0.36 ± 1.01 *(1.00)* | | | ↑ 1.90 ± 1.04 *(0.22)* | ↑ 0.079 ± 0.91  *(1.00)* | | **↓ -3.06 ± 0.98 *(0.027)*** | | |  |
| Campylobacterota | Campylobacteraceae | ↑ 0.11 ± 0.77 *(1.00)* | ↓ -1.87 ± 0.92 *(0.23)* | ↑ 0.62 ± 5.77 *(1.00)* | | | **↓ -4.71 ± 0.84 *(1.13 E-6)*** | ↑ 0.095 ± 0.98 *(1.00)* | | ↑ 2.00 ± 0.85 *(0.10)* | | |  |
| Firmicutes | Acidaminococcaceae | **↓ -4.47 ± 0.79 *(1.08 E-6)*** | - | ↓ -4.76 ± 2.24 *(0.18)* | | | **↑ 10.70 ± 2.63 *(0.00058)*** | ↓ -0.69 ± 0.94 *(1.00)* | | - | | |  |
|  | Anaerovoracaceae | ↓ -0.59 ± 0.50 *(0.84)* | ↑ 1.96 ± 1.37 *(0.68)* | ↑ 0.77 ± 0.55 *(0.75)* | | | **↓ -2.99 ± 0.79 *(0.0014)*** | ↑ 0.30 ± 0.43 *(1.00)* | | ↓ -0.28 ± 0.44 *(0.86)* | | |  |
|  | Butyricicoccaceae | ↓ -0.25 ± 0.43 *(1.00)* | ↓ -0.31 ± 0.57 *(1.00)* | ↓ -0.028 ± 0.43 *(1.00)* | | | **↓ -2.61 ± 0.56 *(7.09 E-5)*** | ↑ 0.32 ± 0.31 *(1.00)* | | ↑ 0.20 ± 0.41 *(0.88)* | | |  |
|  | Enterococcaceae | ↓ -0.57 ± 2.14 *(1.00)* | ↑ 1.16 ± 1.92 *(1.00)* | **↓ -21.36 ± 2.11 *(3.18 E-22)*** | | | **↓ -6.02 ± 1.93 *(0.011)*** | ↓ -4.19 ± 1.92 *(0.80)* | | ↑ 0.26 ± 1.51 *(1.00)* | | |  |
|  | Erysipelatoclostridiaceae | ↑ 1.59 ± 0.73 *(0.27)* | ↓ -0.36 ± 0.79 *(1.00)* | **↓ -2.07 ± 0.48 *(0.00055)*** | | | ↑ 0.51 ± 0.95 *(0.96)* | ↑ 0.62 ± 0.69 *(1.00)* | | ↓ -1.18 ± 0.82 *(0.38)* | | |  |
|  | Erysipelotrichaceae | ↓ -0.56 ± 0.50 *(0.85)* | **↓ -3.04 ± 0.39 *(6.68 E-13)*** | **↓ -1.19 ± 0.35 *(0.011)*** | | | ↓ -0.54 ± 0.66 *(0.87)* | ↓ -0.74 ± 0.45 *(1.00)* | | **↓ -3.42 ± 0.77 *(0.00047)*** | | |  |
|  | [Eubacterium] coprostanoligenes group | ↓ -0.24 ± 0.50 *(1.00)* | ↓ 0.56 ± 0.70 *(1.00)* | ↑ 1.13 ± 0.54 *(0.19)* | | | **↑ 2.98 ± 0.93 *(0.0099)*** | ↓ -0.40 ± 0.47 *(1.00)* | | ↓ -0.26 ± 0.51 *(0.88)* | | |  |
|  | Oscillospiraceae | ↓ -0.16 ± 0.43 *(1.00)* | ↓ 0.36 ± 0.54 *(1.00)* | **↑ 1.41 ± 0.44 *(0.017)*** | | | ↑ 0.78 ± 0.41 *(0.21)* | ↑ 0.35 ± 0.37 *(1.00)* | | ↓ -0.32 ± 0.40 *(0.77)* | | |  |
|  | Peptococcaceae | ↓ -0.34 ± 0.37 *(0.90)* | ↓ -1.18 ± 0.55 *(0.23)* | ↑ 0.39 ± 0.50 *(1.00)* | | | ↓ -0.19 ± 0.47 *(1.00)* | ↑ 0.56 ± 0.35 *(1.00)* | | **↓ -0.85 ± 0.30 *(0.040)*** | | |  |
|  | Unclassified | - | ↑ 0.095 ± 0.61 *(1.00)* | ↑ 0.065 ± 0.40 *(1.00)* | | | **↑ 2.16 ± 0.56 *(0.0012)*** | ↓ -0.58 ± 0.46 *(1.00)* | | ↓ -0.19 ± 0.49 *(0.88)* | | |  |
| **Phylum** | **Family** | **Marker 1 / Washout 1 Log_2_ Fold Change** | | | | | | | | | | |  |
|  |  | **Marker _1_ = Crayon, Marker _2_ = Glitter** | | | | **Marker _1_ = Glitter, Marker _2_ = Crayon** | | | | | | | |
|  |  | **C1** | **C2** | **C3** | | **C4** | | | **C5** | | | **C6** | |
| Firmicutes | Acidaminococcaceae | **↓** -1.72 ± 0.59 *(0.18)* | - | **↓** -0.44 ± 1.79 *(1.00)* | | **↑ 9.49 ± 2.05 *(0.00018)*** | | | **↑** 0.49 ± 0.77 *(1.00)* | | | - | |
| **Phylum** | **Family** | **Marker 1 / Marker 2 Log_2_ Fold Change** | | | | | | | | | | |  |
|  |  | **Marker _1_ = Crayon, Marker _2_ = Glitter** | | | | **Marker _1_ = Glitter, Marker _2_ = Crayon** | | | | | | | |
|  |  | **C1** | **C2** | **C3** | | **C4** | | | **C5** | | | **C6** | |
| Actinobacteriota | Atopobiaceae | ↓ -0.60 ± 0.31 *(0.23)* | ↑ 0.21 ± 0.53 *(1.00)* | ↑ 0.050 ± 0.52 *(1.00)* | | **↑ 3.27 ± 1.12 *(0.020)*** | | | ↓ -0.43 ± 0.29 *(0.39)* | | | ↑ 0.62 ± 0.56 *(0.96)* | |
|  | Bifidobacteriaceae | ↓ -0.55 ± 0.64 *(0.90)* | ↑ 0.99 ± 0.65 *(0.58)* | ↑ 0.15 ± 0.87 *(1.00)* | | **↑ 2.19 ± 0.72 *(0.016)*** | | | ↓ -0.099 ± 0.42 *(1.00)* | | | **↓ -2.22 ± 0.57 *(0.0040)*** | |
| Bacteroidota | Bacteroidaceae | ↑ 0.19 ± 0.30 *(0.99)* | ↑ 0.15 ± 0.34 *(1.00)* | ↑ 1.15 ± 0.43 *(0.074)* | | **↓ -1.75 ± 0.51 *(0.0098)*** | | | ↑ 0.048 ± 0.43 *(1.00)* | | | ↑ 0.049 ± 0.30 *(1.00)* | |
|  | Marinifilaceae | ↑ 0.20 ± 0.42 *(1.00)* | ↓ -0.47 ± 0.51 *(0.87)* | ↑ 0.34 ± 0.48 *(0.89)* | | **↓ -2.15 ± 0.67 *(0.011)*** | | | **↓ -1.85 ± 0.58 *(0.016)*** | | | ↓ -0.63 ± 0.34 *(0.78)* | |
|  | Muribaculaceae | **↑** **1.34 ±** **0.37** ***(0.0068)*** | ↓ -1.26 ± 0.44 *(0.11)* | ↑ 0.095 ± 0.38 *(1.00)* | | **↓ -1.26 ± 0.39 *(0.011)*** | | | **↓ -0.90 ± 0.31 *(0.029)*** | | | ↓ -0.31 ± 0.35 *(0.96)* | |
|  | Rikenellaceae | ↑ 0.79 ± 0.41 *(0.23)* | ↑ 0.26 ± 0.75 *(1.00)* | **↓ -2.42 ± 0.77 *(0.023)*** | | **↓ -2.48 ± 0.77 *(0.011)*** | | | ↓ -1.64 ± 0.69 *(0.087)* | | | ↓ -0.92 ± 0.74 *(0.96)* | |
|  | Tannerellaceae | ↓ -0.037 ± 0.35 *(1.00)* | ↓ -0.52 ± 0.40 *(0.62)* | ↓ -0.15 ± 0.19 *(0.86)* | | **↑ 1.25 ± 0.44 *(0.025)*** | | | ↓ -0.48 ± 0.30 *(0.34)* | | | ↓ -0.23 ± 0.43 *(1.00)* | |
| Campylobacterota | Campylobacteraceae | ↑ 1.23 ± 0.57 *(0.15)* | ↓ -0.26 ± 0.67 *(1.00)* | ↑ 4.62 ± 4.30 *(0.74)* | | **↓ -2.25 ± 0.63 *(0.0079)*** | | | **↓ -2.20 ± 0.74 *(0.026)*** | | | ↓ -0.53 ± 0.64 *(0.96)* | |
| Firmicutes | Acidaminococcaceae | **↓** **-1.68 ± 0.59 *(0.047)*** | - | **↓ -6.35 ± 1.63 *(0.0049)*** | | **↑ 9.46 ± 2.06 *(0.00019)*** | | | **↓ -1.94 ± 0.74 *(0.049)*** | | | - | |
|  | Clostridiaceae | ↑ **3.07 ± 0.64 *(0.00010)*** | ↑ 0.90 ± 0.56 *(0.58)* | ↑ 0.65 ± 0.49 *(0.69)* | | ↑ 1.19 ± 0.54 *(0.098)* | | | **↑ 1.46 ± 0.49 *(0.026)*** | | | - | |
|  | Enterococcaceae | ↓ -3.55 ± 1.62 *(0.15)* | **↓ 5.83 ± 1.39 *(0.0014)*** | ↓ -2.27 ± 1.33 *(0.46)* | | **↓ -3.73 ± 1.45 *(0.046)*** | | | **↓ -6.02 ± 1.45 *(0.00098)*** | | | ↓ -2.59 ± 1.14 *(0.50)* | |
|  | Erysipelatoclostridiaceae | **↑ 1.76 ± 0.56 *(0.021)*** | ↑ 1.06 ± 0.58 *(0.58)* | ↑ 0.19 ± 0.36 *(0.97)* | | ↑ 0.42 ± 0.72 *(0.87)* | | | **↑ 2.30 ± 0.52 *(0.00058)*** | | | ↑ 0.33 ± 0.61 *(1.00)* | |
|  | Erysipelotrichaceae | ↓ -0.58 ± 0.38 *(0.37)* | ↓ -0.48 ± 0.28 *(0.58)* | ↓ -0.67 ± 0.26 *(0.091)* | | ↓ -0.77 ± 0.50 *(0.31)* | | | **↑ 1.18 ± 0.34 *(0.0084)*** | | | ↓ -0.42 ± 0.58 *(0.96)* | |
|  | Eubacteriaceae | **↓ -5.72 ± 1.44 *(0.0019)*** | - | - | | - | | | **-** | | | - | |
|  | [Eubacterium] coprostanoligenes group | ↓ -0.88 ± 0.38 *(0.12)* | ↓ -0.98 ± 0.51 *(0.58)* | **↓ -1.28 ± 0.41 *(0.023)*** | | ↓ -0.21 ± 0.70 *(0.95)* | | | ↓ -0.17 ± 0.36 *(0.94)* | | | ↑ 0.55 ± 0.39 *(0.80)* | |
|  | Streptococcaceae | ↑ 0.56 ± 3.60 *(1.00)* | ↓ -2.16 ± 1.80 *(0.66)* | **↓ -5.96 ± 1.90 *(0.023)*** | | ↓ -5.37 ± 2.37 *(0.087)* | | | **↓ -5.58 ± 1.63 *(0.0084)*** | | | ↓ -1.37 ± 1.80 *(0.96)* | |
| Proteobacteria | Enterobacteriaceae | ↑ 1.63 ± 0.94 *(0.27)* | - | ↑ 1.24 ± 0.95 *(0.69)* | | ↑ 1.41 ± 0.60 *(0.080)* | | | **↑ 1.71 ± 0.60 *(0.029)*** | | | - | |
| **Phylum** | **Family** | **Marker 1 / Washout 2 Log_2_ Fold Change** | | | | | | | | | | |  |
|  |  | **Marker _1_ = Crayon, Marker _2_ = Glitter** | | | | **Marker _1_ = Glitter, Marker _2_ = Crayon** | | | | | | | |
|  |  | **C1** | **C2** | **C3** | | **C4** | | | **C5** | | | **C6** | |
| Actinobacteriota | Atopobiaceae | ↓ -0.29 ± 0.32 *(0.65)* | ↓ -0.12 ± 0.55 *(1.00)* | **↑ 3.30 ± 0.68 *(6.68 E-5)*** | | **↑ 3.46 ± 1.23 *(0.024)*** | | | ↓ -0.45 ± 0.30 *(0.56)* | | | ↑ 0.042 ± 0.59 *(1.00)* | |
|  | Bifidobacteriaceae | ↓ -1.38 ± 0.65 *(0.18)* | ↑ 1.26 ± 0.67 *(0.67)* | ↑ 2.58 ± 0.95 *(0.084)* | | **↑ 4.73 ± 0.80 *(9.70 E-8)*** | | | ↑ 0.16 ± 0.44 *(1.00)* | | | **↑ 1.58 ± 0.59 *(0.042)*** | |
|  | Coriobacteriaceae | **↓ -0.93 ± 0.32 *(0.039)*** | ↑ 0.62 ± 0.34 *(0.67)* | ↑ 0.42 ± 0.28 *(0.85)* | | ↑ 0.44 ± 0.30 *(0.37)* | | | ↓ -0.10 ± 0.23 *(1.00)* | | | **↓ -0.86 ± 0.26 *(0.0090)*** | |
|  | Eggerthellaceae | ↓ -0.61 ± 0.34 *(0.28)* | ↓ -0.064 ± 0.36 *(1.00)* | ↓ -0.13 ± 0.22 *(0.95)* | | ↑ 0.059 ± 0.28 *(1.00)* | | | ↑ 0.15 ± 0.17 *(0.81)* | | | **↓ -0.71 ± 0.23 *(0.015)*** | |
| Bacteroidota | Bacteroidaceae | ↑ 0.61 ± 0.31 *(0.24)* | ↑ 0.44 ± 0.35 *(1.00)* | ↑ 0.98 ± 0.45 *(0.24)* | | **↓ -2.68 ± 0.53 *(7.53 E-6)*** | | | ↑ 0.55 ± 0.45 *(0.73)* | | | **↑ 1.04 ± 0.31 *(0.0083)*** | |
|  | Muribaculaceae | **↑ 1.62 ± 0.39 *(0.0020)*** | ↓ -0.57 ± 0.46 *(1.00)* | ↑ 0.21 ± 0.39 *(0.95)* | | ↓ -0.58 ± 0.41 *(0.38)* | | | ↓ -0.56 ± 0.32 *(0.43)* | | | ↓ -0.55 ± 0.37 *(0.38)* | |
|  | Rikenellaceae | ↑ 0.54 ± 0.43 *(0.49)* | ↑ 1.98 ± 0.81 *(0.36)* | ↓ -1.16 ± 0.80 *(0.85)* | | **↑ 3.54 ± 0.82 *(0.00018)*** | | | ↓ -0.80 ± 0.72 *(0.73)* | | | **↓ -2.87 ± 0.77 *(0.0031)*** | |
| Campylobacterota | Campylobacteraceae | **↑ 2.16 ± 0.60 *(0.0047)*** | ↓ -0.80 ± 0.70 *(1.00)* | ↑ 4.66 ± 4.48 *(0.85)* | | **↓ -4.55 ± 0.65 *(1.64 E-10)*** | | | ↓ -0.15 ± 0.77 *(1.00)* | | | ↑ 0.69 ± 0.67 *(0.56)* | |
| Firmicutes | Acidaminococcaceae | **↓ -1.78 ± 0.61 *(0.039)*** | - | **↓ -5.47 ± 1.69 *(0.020)*** | | **↑ 8.49 ± 2.10 *(0.00046)*** | | | **↓ -5.11 ± 0.76 *(1.58 E-9)*** | | | - | |
|  | Butyricicoccaceae | ↑ 0.26 ± 0.34 *(0.77)* | ↓ -0.073 ± 0.44 *(1.00)* | ↓ -0.33 ± 0.34  *(0.85)* | | **↓ -2.36 ± 0.44 *(1.87 E-6)*** | | | ↓ -0.27 ± 0.25 *(0.73)* | | | ↓ -0.52 ± 0.33 *(0.35)* | |
|  | Enterococcaceae | ↓ -3.15 ± 1.68 *(0.25)* | ↑ 3.08 ± 1.47 *(0.59)* | ↑ 0.54 ± 1.38 *(1.00)* | | **↓ -4.00 ± 1.51 *(0.034)*** | | | **↓ -5.34 ± 1.51 *(0.0093)*** | | | ↓ -0.99 ± 1.19 *(0.68)* | |
|  | Erysipelatoclostridiaceae | ↑ 0.63 ± 0.58 *(0.56)* | ↑ 0.37 ± 0.60 *(1.00)* | ↓ -0.38 ± 0.38 *(0.85)* | | **↓ -1.83 ± 0.74 *(0.049)*** | | | **↓ 1.67 ± 0.54 *(0.028)*** | | | ↓ -0.39 ± 0.64 *(0.80)* | |
|  | Erysipelotrichaceae | **↓ -1.45 ± 0.40 *(0.0047)*** | ↓ -0.16 ± 0.29 *(1.00)* | **↓ -1.06 ± 0.27 *(0.0029)*** | | **↓ -1.58 ± 0.52 *(0.013)*** | | | ↑ 0.50 ± 0.35 *(0.62)* | | | **↓ -2.52 ± 0.61 *(0.0015)*** | |
|  | Eubacteriaceae | **↓ -5.46 ± 1.49 *(0.0047)*** | - | - | | - | | | - | | | - | |
|  | [Eubacterium] coprostanoligenes group | ↓ -0.66 ± 0.40 *(0.32)* | ↓ -0.32 ± 0.53 *(1.00)* | ↑ 0.026 ± 0.43 *(1.00)* | | **↑ 2.67 ± 0.73 *(0.0020)*** | | | ↓ -0.42 ± 0.37 *(0.73)* | | | ↓ -0.11 ± 0.40  *(0.93)* | |
|  | Peptostreptococcaceae | ↓ -0.079 ± 0.37  *(1.00)* | ↑ 0.37 ± 0.38 *(1.00)* | ↑ 0.15 ± 0.26 *(0.95)* | | **↓ -1.04 ± 0.38 *(0.030)*** | | | ↑ 0.53 ± 0.25 *(0.25)* | | | - | |
|  | Selenomonadaceae | ↓ -0.72 ± 0.58 *(0.49)* | ↓ -0.039 ± 0.64 *(1.00)* | ↑ 1.93 ± 0.82 *(0.19)* | | ↓ -0.38 ± 0.76 *(0.91)* | | | ↑ 0.60 ± 0.62 *(0.81)* | | | **↑ 2.07 ± 0.56 *(0.0031)*** | |
|  | Unclassified | ↑ 0.35 ± 0.49 *(0.80)* | ↓ -0.58 ± 0.47 *(1.00)* | ↓ -0.25 ± 0.31 *(0.95)* | | **↑ 1.13 ± 0.44 *(0.041)*** | | | **↓ -1.06 ± 0.36 *(0.041)*** | | | ↓ -0.91 ± 0.39 *(0.079)* | |
| Fusobacteriota | Fusobacteriaceae | ↑ 0.67 ± 0.44 *(0.36)* | ↑ 1.80 ± 0.57 *(0.082)* | ↑ 0.51 ± 0.44 *(0.85)* | | **↓ -1.94 ± 0.63 *(0.013)*** | | | **↑ 1.46 ± 0.42 *(0.0093)*** | | | - | |
| Proteobacteria | Sutterellaceae | ↑ 0.44 ± 0.47 *(0.65)* | ↓ -0.088 ± 0.52 *(1.00)* | ↑ 0.59 ± 0.45 *(0.85)* | | ↓ -0.90 ± 0.53 *(0.27)* | | | ↑ 1.10 ± 0.57 *(0.33)* | | | **↑ 1.02 ± 0.29 *(0.0057)*** | |
| **Phylum** | **Family** | **Washout 1 / Marker 2 Log_2_ Fold Change** | | | | | | | | | | |  |
|  |  | **Marker _1_ = Crayon, Marker _2_ = Glitter** | | | | **Marker _1_ = Glitter, Marker _2_ = Crayon** | | | | | | | |
|  |  | **C1** | **C2** | **C3** | | **C4** | | | **C5** | | | **C6** | |
| Actinobacteriota | Atopobiaceae | **↓ -0.92 ± 0.31 *(0.044)*** | ↓ -0.92 ± 0.88 *(1.00)* | ↓ -0.46 ± 0.53 *(0.82)* | | ↑ 1.04 ± 1.18 *(0.66)* | | | ↓ -0.29 ± 0.29 *(0.84)* | | | ↑ 1.18 ± 0.56 *(0.72)* | |
|  | Bifidobacteriaceae | ↑ 0.70 ± 0.62 *(0.76)* | ↓ 0.52 ± 1.01 *(1.00)* | ↓ -1.30 ± 0.88 *(0.46)* | | **↑ 2.32 ± 0.72 *(0.016)*** | | | ↑ 0.25 ± 0.42 *(1.00)* | | | **↑ 2.12 ± 0.57 *(0.0088)*** | |
| Bacteroidota | Bacteroidaceae | ↑ 0.20 ± 0.30 *(1.00)* | ↓ 0.53 ± 0.53 *(1.00)* | ↑ 0.67 ± 0.43 *(0.44)* | | **↓ -1.82 ± 0.51 *(0.0077)*** | | | ↓ -0.064 ± 0.43 *(1.00)* | | | ↓ -0.17 ± 0.30 *(1.00)* | |
|  | Marinifilaceae | ↓ -0.073 ± 0.42 *(1.00)* | ↓ -0.61 ± 0.80 *(1.00)* | ↑ 0.59 ± 0.48 *(0.53)* | | **↓ -2.38 ± 0.68 *(0.0077)*** | | | ↓ -1.33 ± 0.57 *(0.12)* | | | ↓ -0.46 ± 0.34 *(1.00)* | |
|  | Rikenellaceae | **↑** 0.94 ± 0.41 *(0.20)* | ↓ -1.24 ± 1.22 *(1.00)* | ↓ -0.83 ± 0.76 *(0.65)* | | **↓ -2.89 ± 0.77 *(0.0077)*** | | | **↓ -2.29 ± 0.69 *(0.021)*** | | | ↑ 0.40 ± 0.74 *(1.00)* | |
|  | Tannerellaceae | ↑ 0.099 ± 0.35 *(1.00)* | ↑ 0.16 ± 0.62 *(1.00)* | ↓ -0.14 ± 0.19 *(0.83)* | | **↓ -1.40 ± 0.44 *(0.016)*** | | | ↓ -0.85 ± 0.30 *(0.054)* | | | ↓ -0.16 ± 0.43 *(1.00)* | |
| Firmicutes | Acidaminococcaceae | ↑ 0.042 ± 0.59 *(1.00)* | - | **↓ -5.91 ± 1.63 *(0.014)*** | | ↓ -0.027 ± 2.17 *(1.00)* | | | **↓ -2.44 ± 0.75 *(0.021)*** | | | - | |
|  | Clostridiaceae | **↑ 2.29 ± 0.64 *(0.0093)*** | ↑ 0.69 ± 0.87 *(1.00)* | ↑ 0.68 ± 0.49 *(0.47)* | | ↑ 1.22 ± 0.54 *(0.13)* | | | **↑ 1.56 ± 0.49 *(0.021)*** | | | - | |
|  | Enterococcaceae | ↓ -1.53 ± 1.62 *(0.91)* | ↓ -6.43 ± 2.17 *(0.14)* | ↑ 2.20 ± 0.95 *(0.16)* | | ↓ -2.87 ± 1.45 *(0.18)* | | | **↓ -6.73 ± 1.46 *(0.00022)*** | | | ↓ -1.13 ± 1.14 *(1.00)* | |
|  | Erysipelatoclostridiaceae | **↑ 1.94 ± 0.56  *(0.0093)*** | ↓ -0.52 ± 0.90 *(1.00)* | ↓ 0.10 ± 0.36 *(1.00)* | | ↑ 1.05 ± 0.72 *(0.36)* | | | ↑ 1.27 ± 0.52 *(0.11)* | | | ↑ 0.32 ± 0.61 *(1.00)* | |
|  | Eubacteriaceae | **↓ -5.67 ± 1.43 *(0.0042)*** | - | - | | - | | | - | | | - | |
|  | Streptococcaceae | ↑ 0.79 ± 3.60 *(1.00)* | ↓ -4.43 ± 3.03 *(1.00)* | **↓ -6.21 ± 1.92 *(0.030)*** | | ↓ -5.62 ± 2.37 *(0.12)* | | | ↓ -3.95 ± 1.55 *(0.10)* | | | ↓ -2.86 ± 1.89 *(1.00)* | |
| **Phylum** | **Family** | **Washout 1 / Washout 2 Log_2_ Fold Change** | | | | | | | | | | |  |
|  |  | **Marker _1_ = Crayon, Marker _2_ = Glitter** | | | | **Marker _1_ = Glitter, Marker _2_ = Crayon** | | | | | | |  |
|  |  | **C1** | **C2** | | **C3** | **C4** | | | **C5** | | **C6** | |  |
| Actinobacteriota | Atopobiaceae | ↓ -0.62 ± 0.32 *(0.34)* | ↓ -1.27 ± 0.89 *(1.00)* | | **↑ 2.78 ± 0.68 *(0.0025)*** | ↑ 1.22 ± 1.28 *(0.53)* | | | ↓ -0.31 ± 0.30 *(0.76)* | | ↑ 0.60 ± 0.58 *(0.53)* | |  |
|  | Bifidobacteriaceae | ↓ -0.12 ± 0.63 *(0.98)* | ↑ 0.80 ± 1.02 *(1.00)* | | ↑ 1.12 ± 0.96 *(0.76)* | **↑ 4.86 ± 0.80 *(5.25 E-8)*** | | | ↑ 0.51 ± 0.44 *(0.66)* | | ↑ 1.47 ± 0.59 *(0.11)* | |  |
|  | Coriobacteriaceae | **↓ -1.07 ± 0.32 *(0.020)*** | ↓ -0.041 ± 0.52 *(1.00)* | | ↑ 0.18 ± 0.28 *(1.00)* | ↑ 0.36 ± 0.30 *(0.39)* | | | ↓ -0.29 ± 0.23 *(0.66)* | | **↓ -0.92 ± 0.26 *(0.012)*** | |  |
| Bacteroidaceae | Bacteroidaceae | ↑ 0.62 ± 0.31 *(0.34)* | ↓ 0.83 ± 0.54 *(1.00)* | | ↑ 0.49 ± 0.45 *(0.78)* | **↓ -2.74 ± 0.53 *(3.05 E-6)*** | | | ↑ 0.44 ± 0.45 *(0.76)* | | ↑ 0.81 ± 0.31 *(0.10)* | |  |
|  | Rikenellaceae | ↑ 0.70 ± 0.43 *(0.43)* | ↓ 0.46 ± 1.26 *(1.00)* | | ↑ 0.42 ± 0.80 *(1.00)* | **↑ 3.12 ± 0.83 *(0.00098)*** | | | ↓ -1.45 ± 0.72 *(0.38)* | | ↓ -1.53 ± 0.76 *(0.16)* | |  |
| Campylobacterota | Campylobacteraceae | ↑ 0.70 ± 0.61 *(0.71)* | ↓ -0.30 ± 1.06 *(1.00)* | | 0.00 ± 4.54 *(1.00)* | **↓ -3.73 ± 0.64 *(1.05 E-7)*** | | | ↑ 1.34 ± 0.77 *(0.50)* | | ↑ 0.76 ± 0.67 *(0.49)* | |  |
| Firmicutes | Acidaminococcaceae | ↓ -0.059 ± 0.61 *(1.00)* | - | | ↓ -5.03 ± 1.69 *(0.072)* | ↑ 1.00 ± 2.22 *(0.80)* | | | **↓ -5.60 ± 0.78 *(3.74 E-11)*** | | - | |  |
|  | Butyricicoccaceae | ↑ 0.25 ± 0.34 *(0.97)* | ↑ 0.077 ± 0.66  *(1.00)* | | ↑ 0.22 ± 0.33 *(1.00)* | **↓ -2.63 ± 0.44 *(6.66 E-8)*** | | | ↓ -0.14 ± 0.25 *(0.93)* | | ↓ -0.47 ± 0.33 *(0.37)* | |  |
|  | Enterococcaceae | ↓ -1.13 ± 1.68 *(0.97)* | ↑ 2.47 ± 2.22 *(1.00)* | | ↓ -0.0067 ± 1.38 *(1.00)* | ↓ -3.13 ± 1.51 *(0.11)* | | | **↓ -6.05 ± 1.52 *(0.0018)*** | | ↑ 0.46 ± 1.19 *(0.97)* | |  |
|  | Erysipelotrichaceae | **↓ -1.50 ± 0.40 *(0.0070)*** | ↓ -0.35 ± 0.45 *(1.00)* | | ↓ -0.48 ± 0.27 *(0.68)* | ↓ -0.71 ± 0.52 *(0.30)* | | | ↓ -0.48 ± 0.35 *(0.62)* | | **↓ -2.09 ± 0.61 *(0.012)*** | |  |
|  | Eubacteriaceae | **↓ -5.41 ± 1.48 *(0.0070)*** | - | | - | - | | | - | | - | |  |
|  | [Eubacterium] coprostanoligenes group | ↓ -0.35 ± 0.40 *(0.87)* | ↓ -0.30 ± 0.81 *(1.00)* | | ↑ 0.17 ± 0.43 *(1.00)* | ↑ 2.75 ± 0.73 *(0.0010)* | | | ↓ -0.73 ± 0.37 *(0.38)* | | ↓ -0.28 ± 0.40 *(0.77)* | |  |
|  | Peptostreptococcaceae | ↓ -0.22 ± 0.37 *(0.97)* | ↓ -0.17 ± 0.58 *(1.00)* | | ↑ 0.10 ± 0.26 *(1.00)* | **↓ -1.02 ± 0.38 *(0.037)*** | | | ↑ 0.22 ± 0.25 *(0.76)* | | - | |  |
|  | Veillonellaceae | ↑ 0.78 ± 0.38 *(0.34)* | ↓ -0.55 ± 0.66 *(1.00)* | | ↓ -0.36 ± 0.27 *(0.76)* | **↑ 1.51 ± 0.59 *(0.045)*** | | | ↓ -0.027 ± 0.27 *(1.00)* | | ↓ -0.53 ± 0.45 *(0.47)* | |  |
| Fusobacteriota | Fusobacteriaceae | ↑ 0.60 ± 0.44 *(0.61)* | ↑ 1.64 ± 0.87 *(1.00)* | | ↑ 0.16 ± 0.44 *(1.00)* | **↓ -2.54 ± 0.63 *(0.00047)*** | | | **↑ 1.34 ± 0.42 *(0.026)*** | | - | |  |
| **Phylum** | **Family** | **Marker 2 / Washout 2 Log_2_ Fold Change** | | | | | | | | | | |  |
|  |  | **Marker _1_ = Crayon, Marker _2_ = Glitter** | | | | **Marker _1_ = Glitter, Marker _2_ = Crayon** | | | | | | |  |
|  |  | **C1** | **C2** | | **C3** | **C4** | | | **C5** | | **C6** | |  |
| Actinobacteriota | Atopobiaceae | ↑ 0.30 ± 0.32 *(1.00)* | ↓ -0.34 ± 0.57 *(0.99)* | | **↑ 3.25 ± 0.68 *(0.00010)*** | ↑ 0.18 ± 1.36 *(1.00)* | | | ↓ -0.021 ± 0.30 *(1.00)* | | ↓ -0.58 ± 0.60 *(0.60)* | |  |
|  | Bifidobacteriaceae | ↓ -0.83 ± 0.64 *(0.87)* | ↑ 0.27 ± 0.70 *(1.00)* | | ↑ 2.43 ± 0.95 *(0.18)* | **↑ 2.54 ± 0.80 *(0.010)*** | | | ↑ 0.26 ± 0.44 *(1.00)* | | ↓ -0.64 ± 0.61 *(0.55)* | |  |
|  | Coriobacteriaceae | ↓ -0.47 ± 0.32 *(0.86)* | ↑ 0.45 ± 0.35 *(0.82)* | | ↑ 0.67 ± 0.28 *(0.24)* | ↓ -0.058 ± 0.30 *(1.00)* | | | ↓ -0.42 ± 0.23 *(0.36)* | | **↓ -0.83 ± 0.26 *(0.027)*** | |  |
| Bacteriodota | Bacteroidaceae | ↑ 0.42 ± 0.31 *(0.87)* | ↑ 0.29 ± 0.36 *(0.99)* | | ↓ -0.17 ± 0.45 *(1.00)* | ↓ -0.92 ± 0.53 *(0.25)* | | | ↑ 0.50 ± 0.45 *(0.83)* | | **↑ 0.99 ± 0.31** ***(0.027)*** | |  |
|  | Marinifilaceae | ↑ 0.89 ± 0.44 *(0.55)* | ↑ 0.83 ± 0.55 *(0.81)* | | ↑ 0.038 ± 0.50 *(1.00)* | **↑ 1.88 ± 0.71 *(0.030)*** | | | ↑ 1.42 ± 0.60 *(0.19)* | | ↑ 0.77 ± 0.36 *(0.15)* | |  |
|  | Rikenellaceae | ↓ -0.24 ± 0.43 *(1.00)* | ↑ 1.71 ± 0.83 *(0.81)* | | ↑ 1.26 ± 0.80 *(0.60)* | **↑ 6.02 ± 0.82 *(1.76 E-11)*** | | | ↑ 0.84 ± 0.72 *(0.83)* | | ↓ -1.94 ± 0.77 *(0.070)* | |  |
|  | Tannerellaceae | ↑ 0.54 ± 0.36 *(0.86)* | ↑ 0.70 ± 0.43 *(0.81)* | | ↑ 0.26 ± 0.20 *(0.60)* | **↑ 1.28 ± 0.46** ***(0.026)*** | | | ↑ 0.77 ± 0.31 *(0.19)* | | ↓ -0.39 ± 0.45 *(0.66)* | |  |
| Campylobacterota | Campylobacteraceae | ↑ 0.93 ± 0.61 *(0.86)* | ↓ -0.54 ± 0.72 *(0.99)* | | 0.00 ± 4.54 *(1.00)* | **↓ -2.29 ± 0.64 *(0.0025)*** | | | ↑ 2.05 ± 0.77 *(0.14)* | | ↑ 1.22 ± 0.67 *(0.21)* | |  |
| Firmicutes | Acidaminococcaceae | ↓ -0.10 ± 0.61 *(1.00)* | - | | ↑ 0.87 ± 1.51 *(1.00)* | ↓ -0.97 ± 2.22 *(1.00)* | | | **↓ -3.16 ± 0.75 *(0.0014)*** | | - | |  |
|  | Butyricicoccaceae | ↑ 0.32 ± 0.34 *(1.00)* | ↓ -0.39 ± 0.45 *(0.99)* | | ↓ -0.68 ± 0.34 *(0.37)* | **↓ -2.09 ± 0.44 *(6.13 E-5)*** | | | ↓ -0.46 ± 0.25 *(0.36)* | | ↓ -0.051 ± 0.33 *(1.00)* | |  |
|  | Clostridiaceae | ↓ -2.07 ± 0.67 *(0.11)* | ↓ -0.81 ± 0.60 *(0.82)* | | ↓ -0.36 ± 0.51 *(1.00)* | **↓ -2.52 ± 0.56 *(0.00013)*** | | | ↓ -1.15 ± 0.51 *(0.23)* | | - | |  |
|  | Enterococcaceae | ↑ 0.39 ± 1.68 *(1.00)* | **↑ 8.91 ± 1.51 *(2.01 E-7)*** | | ↑ 2.82 ± 1.38 *(0.37)* | ↓ -0.26 ± 1.51 *(1.00)* | | | ↑ 0.67 ± 1.51 *(1.00)* | | ↑ 1.60 ± 1.19 *(0.41)* | |  |
|  | Erysipelotrichaceae | ↓ -0.86 ± 0.39 *(0.54)* | ↑ 0.32 ± 0.30 *(0.85)* | | ↓ -0.38 ± 0.27 *(0.60)* | ↓ -0.80 ± 0.52 *(0.30)* | | | ↓ -0.68 ± 0.35 *(0.36)* | | **↓ -2.09 ± 0.61 *(0.025)*** | |  |
|  | Erysipelatoclostridiaceae | ↓ -1.13 ± 0.58 *(0.55)* | ↓ -0.69 ± 0.62 *(0.83)* | | ↓ -0.58 ± 0.38 *(0.60)* | **↓ -2.26 ± 0.74 *(0.012)*** | | | ↓ -0.62 ± 0.54 *(0.83)* | | ↓ -0.72 ± 0.64 *(0.52)* | |  |
|  | [Eubacterium] coprostanoligenes group | ↑ 0.22 ± 0.39 *(1.00)* | ↑ 0.65 ± 0.55 *(0.82)* | | ↑ 1.31 ± 0.43 *(0.058)* | **↑ 2.88 ± 0.73 *(0.00084)*** | | | ↓ -0.24 ± 0.37 *(1.00)* | | ↓ -0.67 ± 0.41 *(0.28)* | |  |
|  | Peptostreptococcaceae | ↓ -0.17 ± 0.37 *(1.00)* | ↓ -0.013 ± 0.39 *(1.00)* | | ↑ 0.33 ± 0.26 *(0.60)* | **↓ -1.58 ± 0.38 *(0.00051)*** | | | ↓ 0.028 ± 0.25 *(1.00)* | | - | |  |
|  | Veillonellaceae | ↑ 0.0095 ± 0.38 *(1.00)* | ↓ -0.21 ± 0.45 *(1.00)* | | ↑ 0.0091 ± 0.27 *(1.00)* | **↑ 1.61 ± 0.59 *(0.028)*** | | | ↓ -0.056 ± 0.27 *(1.00)* | | ↓ -0.29 ± 0.45 *(0.81)* | |  |
| Fusobacteriota | Fusobacteriaceae | ↓ -0.099 ± 0.44 *(1.00)* | ↑ 1.06 ± 0.59 *(0.81)* | | ↓ -0.20 ± 0.44 *(1.00)* | **↓ -1.93 ± 0.63 *(0.012)*** | | | ↑ 1.32 ± 0.42 *(0.045)* | | - | |  |
| Proteobacteria | Enterobacteriaceae | ↑ 0.65 ± 0.99 *(1.00)* | - | | ↓ -0.48 ± 0.99 *(1.00)* | **↓ -2.45 ± 0.63 *(0.00084)*** | | | ↓ -0.52 ± 0.63 *(0.93)* | | - | |  |
| Log2 fold changes ± standard error and p values (*parentheses*) for differentially abundant families **(bold)** identified across experimental phases using Deseq2. p-values are adjusted for multiple comparisons using a Benjamini-Hochberg posthoc correction. Arrows indicate the direction of the fold change, where ↑ indicates that the microbial abundance is higher in the numerator experimental phases versus in the denominator experimental phase, and ↓ indicates that microbial abundance is lower in the numerator experimental phase versus in the denominator experimental phase. “ - ” indicates the family was not present in the given comparison.  ^a.^ Amplicon Sequencing Variant was “unclassified” at the family level for 5 of 6 cats. For Cat 2, the amplicon sequencing variant classified as family Sutterellaceae.  **Abbreviations:** C1 = Cat 1; C2 = Cat 2; C3 = Cat 3; C4 = Cat 4; C5 = Cat 5; C6 = Cat 6. | | | | | | | | | | | | |  |
